# *Lactobacillus ruminis* strains cluster according to their mammalian gut source

**DOI:** 10.1186/s12866-015-0403-y

**Published:** 2015-04-01

**Authors:** Michelle M O’ Donnell, Hugh Michael B Harris, Denise B Lynch, Reynolds Paul Ross, Paul W O’Toole

**Affiliations:** Teagasc Food Research Centre, Moorepark, Fermoy, Co. Cork, Ireland; School of Microbiology & Alimentary Pharmabiotic Centre, University College Cork, Cork, Ireland; College of Science, Engineering and Food Science (SEFS), University College Cork, Cork, Ireland; School of Microbiology, Food Science Building, University College Cork, Cork, Ireland

**Keywords:** *Lactobacillus ruminis*, *Lactobacillus*, Motility, Prebiotics, RNA-seq

## Abstract

**Background:**

*Lactobacillus ruminis* is a motile *Lactobacillus* that is autochthonous to the human gut, and which may also be isolated from other mammals. Detailed characterization of *L. ruminis* has previously been restricted to strains of human and bovine origin. We therefore sought to expand our bio-bank of strains to identify and characterise isolates of porcine and equine origin by comparative genomics.

**Results:**

We isolated five strains from the faeces of horses and two strains from pigs, and compared their motility, biochemistry and genetic relatedness to six human isolates and three bovine isolates including the type strain 27780^T^. Multilocus sequence typing analysis based on concatenated sequence data for six individual loci separated the 16 *L. ruminis* strains into three clades concordant with human, bovine or porcine, and equine sources. Sequencing the genomes of four additional strains of human, bovine, equine and porcine origin revealed a high level of genome synteny, independent of the source animal. Analysis of carbohydrate utilization, stress survival and technological robustness in a combined panel of sixteen *L. ruminis* isolates identified strains with optimal survival characteristics suitable for future investigation as candidate probiotics. Under laboratory conditions, six human isolates of *L. ruminis* tested were aflagellate and non-motile, whereas all 10 strains of bovine, equine and porcine origin were motile. Interestingly the equine and porcine strains were hyper-flagellated compared to bovine isolates, and this hyper-flagellate phenotype correlated with the ability to swarm on solid medium containing up to 1.8% agar. Analysis by RNA sequencing and qRT-PCR identified genes for the biosynthesis of flagella, genes for carbohydrate metabolism and genes of unknown function that were differentially expressed in swarming cells of an equine isolate of *L. ruminis*.

**Conclusions:**

We suggest that *Lactobacillus ruminis* isolates have potential to be used in the functional food industry. We have also identified a MLST scheme able to distinguish between strains of *L. ruminis* of different origin. Genes for non-digestible oligosaccharide metabolism were identified with a putative role in swarming behaviour.

**Electronic supplementary material:**

The online version of this article (doi:10.1186/s12866-015-0403-y) contains supplementary material, which is available to authorized users.

## Background

*Lactobacillus ruminis* is a commensal species in the gastrointestinal tract (GIT) of humans [1-4] and other mammals including ruminants [5,6], monogastric fermentors [7-11], hindgut fermentors [12,13], additional mammals [14] and birds [15,16]. *L. ruminis* was first identified in 1961 and classified as *Catenabacterium catenaforme* [17], but was re-classified in 1973 when Sharpe *et al*. characterised three isolates from the steer rumen [5]. *L. ruminis* has been described as an autochthonous species present in the GIT of humans [3,18]. Previous studies have noted that *L. ruminis* has potential immunomodulatory properties [19,20] as well as a possible role in suppressing antibiotic-resistant pathogens [21].

Habitual diet and carbohydrate content could restrict the ability of a given *Lactobacillus* species to colonize the GIT [22]. In an attempt to rationalize why *L. ruminis* might be variably present in different species and indeed in different animals of the same species in different studies, we previously characterised the fermentation properties of human and bovine *L. ruminis* isolates [23]. Comparison of the fermentation profiles and genome sequences of ATCC 25644 (human isolate) and ATCC 27782 (bovine isolate) identified the enzymes and pathways that *L. ruminis* used to ferment carbohydrates including α-galactoside, β-galactoside, α-glucoside, β-glucoside and β-fructofuranoside [23,24]. We identified the degree of polymerisation (DP) as an important factor in the fermentability of the carbohydrates tested, with high-DP carbohydrates not being fermented, while carbohydrates with DP of ≤ 10 were readily fermented. The prebiotic fructooligosaccharide (FOS) was fermented by all of the human isolates tested, but the bovine isolate ATCC 27782 failed to ferment this carbohydrate, which was attributed to the absence of beta-fructofuranosidase [23,24].

Being a member of a phylogenetic clade of the lactobacilli that includes the probiotic species *L. salivarius* [19], *L. ruminis* itself is an interesting prospect for development as a probiotic. As outlined above, its autochthonous nature and ability to produce flagella might confer unique probiotic properties. However, for commercialisation of LAB cultures as starter cultures, food ingredients or probiotics, it is necessary to optimize the survival of the strain during exposure to high salt concentrations, aerobic conditions and other processing stages. Thus, *Lactobacillus* species such as *L. plantarum* are often used as functional ingredients due to their innate resistance to harsh conditions [25]. Common criteria required of probiotic cultures include resistance to intestinal conditions (gastric acidity and bile salts), beta-galactosidase activity and known carbohydrate utilization for optimized culture production. Bile resistance is also desirable, because bile levels in the GIT range between 0.3-0.5% [26], which exerts an antimicrobial effect [27]. Antibiotic susceptibility and resistance are additional considerations in assessing suitability for use in human or animal feed, and such resistances can be comprehensively investigated by genome sequencing [28]. In general, *Lactobacillus* species are resistant to the aminoglycoside family of antibiotics, which includes streptomycin, neomycin, gentamycin and kanamycin, and susceptible to broad-spectrum antibiotics such as chloramphenicol and rifampicin [29]. An additional consideration for LAB administration is the vehicle to be used. Due to an increasing frequency of lactose intolerance among consumers, there has been a recent move away from the use of dairy products as vehicles for probiotics [30]. Non-dairy products such as fermented vegetable products represent viable alternatives. However, this places limits and technological stresses on the probiotic cultures in these products due to the salt concentration, aerobic environment and varied temperatures [31].

Motility has previously been described for the bovine isolates of *L. ruminis* [5,19]. Motility has also been identified in other lactobacilli, but was poorly characterised [32-35]. We previously noted that *L. ruminis* was the only *Lactobacillus* isolated from mammals that had a motile phenotype [19]. Transcriptomic analysis of a non-motile human *L. ruminis* isolate and a motile bovine isolate revealed a significant up-regulation of genes in the motility locus of the latter [19]. Many, but not all, bacterial flagellin proteins are recognised by toll-like receptor 5 (TLR5) [36] and we showed that the *L. ruminis* flagellin protein induced IL-8 secretion in several cell lines [19]. We also hypothesized that the aflagellate phenotype of all tested *L. ruminis* strains from human sources might be an inflammation-avoidance mechanism, or selection outcome [19], although a recent report of RNA-seq data from a human *L. ruminis* isolate indicated expression of motility genes and genes in the cellobiose operon in CDP4 medium [37].

Motility in bacterial cells can be classified as swimming or swarming. Swimming refers to classical flagellum-mediated propulsion in liquid, while swarming is flagellum-driven movement over a solid surface like agar [38-40]. Swarming species each appear to have their own “unique” mechanism for facilitating swarming [41]. The FliL protein, part of the type-III flagellar export system and the switch complex, has been shown to be a key component for swarming motility in Salmonella [42]. The swarming ability of bacteria is often cell-density dependent and involves hyper-flagellation, cell differentiation and the possible involvement of polysaccharides and bio-surfactants [40,43]. The addition of bio-surfactants like Tween 80 has been shown to facilitate swarming and to aid in the ease of measurement of a swarm halo [44]. Culture on agar plates of the microbiota of starch-fed horses previously identified the presence of swarming *L. ruminis* [12,13]. Transcriptional analysis has been applied to study *L. ruminis* swimming motility in response to carbohydrate availability [37], but swarming in *L. ruminis* has not been studied using genomic approaches.

In this study, we aimed to expand the knowledge base for *L. ruminis* by isolating an enlarged panel of strains, drawing on two additional mammals known to harbour this species in the GIT - horses and pigs. We used genetic typing, biochemical testing and genome sequencing to examine strain relatedness as a function of host animal, and we identified genes correlated with the ability of certain strains to display swarming motility.

## Results

### *L. ruminis* strain isolation and identification

Faecal samples from 4 sows, 4 weanlings and 10 horses were serially diluted (10^−8^) and plated to isolate *L. ruminis*. Two hundred and fifty-nine colonies in total from the sow and weanling samples and 77 from horses were sub-cultured into MRS broth and grown anaerobically at 37°C for further phenotypic screening. Seventy percent (63/90) of the plates harboured swarming colonies. Isolation of single colonies from the equine faecal samples was difficult due to the abundance of swarming bacteria that covered the plates. A similar level of colony swarming was noted during analysis of faecal samples from Swedish racehorses [13].

In a previous study we established the carbohydrate fermentation profile for *L. ruminis* [23]. This profile was exploited here to screen the candidate *L. ruminis* isolates from the stocked *Lactobacillus* strains of porcine and equine origin. From the 259 porcine isolates, 57 were identified as having a similar fermentation profile to that of *L. ruminis*. Morphology and Gram staining of the 57 isolates was investigated, with 25 of the isolates being Gram positive, catalase negative rods. A similar method was used in the isolation of the 77 equine strains, where the morphological and phenotypic screening reduced the number of isolates to 24.

Genomic DNA from the 49 potential *L. ruminis* isolates was extracted and screened using an *L. ruminis*-specific 16S rRNA gene primer pair (Additional file 1). This reduced the number of isolates to 14 (6 porcine and 8 equine). The 16S rRNA gene was sequenced for six porcine isolates and eight equine isolates (Table 1). Two porcine and 5 equine isolates were identified as *L. ruminis*. A 16S rRNA phylogenetic tree was created from the 16S rRNA gene sequences of the *L. ruminis* isolates and selected closely related species in the *L. salivarius* clade (Additional file 2). Although their 16S rRNA gene sequences were almost identical, the *L. ruminis* isolates were arranged into 3 clades. The human and porcine isolates clustered together and formed a shared clade based on identical 16S rRNA gene sequences except for a polymorphism at residue 5 in the sequence from strain S23. The sequences from the equine strains formed another clade, driven by an A residue (rather than a G residue) at position 490. The bovine isolates formed a separate clade based upon their sharing a T residue at position 206, compared to other strains that had a C residue in this position.

**Table 1:** **Bacterial strains used in this study**

**Origin**	**Strain**	Species identity*	**Source or Reference**
Human	L5	*L. ruminis*	(4)
Human	S21	*L. ruminis*	(4)
Human	S23	*L. ruminis*	(4)
Human	S36	*L. ruminis*	(4)
Human	S38	*L. ruminis*	(4)
Human	ATCC 25644	*L. ruminis*	(1)
Bovine	ATCC 27780^T^	*L. ruminis*	(3)
Bovine	ATCC 27781	*L. ruminis*	(3)
Bovine	ATCC 27782	*L. ruminis*	(3)
Porcine	DPC 6830	*L. ruminis*	This study
Porcine	DPC 6831	*L. ruminis*	This study
Equine	DPC 6832	*L. ruminis*	This study
Equine	DPC 6836	*L. ruminis*	This study
Equine	DPC 6833	*L. ruminis*	This study
Equine	DPC 6834	*L. ruminis*	This study
Equine	DPC 6835	*L. ruminis*	This study
Porcine	AR110	*Streptococcus alactolyticus*	This study
Porcine	AR114	*Lactobacillus acidophilus*	This study
Porcine	WR215	*Lactobacillus johnsonii*	This study
Porcine	W308	*Lactobacillus amylovorus*	This study
Porcine	W312	*Lactobacillus amylovorus*	This study
Equine	4R51	*Streptococcus equinus*	This study
Equine	5R4S1	*Streptococcus equinus*	This study
Equine	5R6S1	*Streptococcus equinus*	This study

*Strains isolated and identified in this study, by BLASTN analysis of 16S rRNA gene amplicons.

### Multilocus sequence typing of *L. ruminis*

To further examine *L. ruminis* strain relatedness as a function of source animal, six loci were amplified and sequenced, and MLST allelic profiles were assigned based on sequence lengths ranging from 616 bp to 765 bp. The 16 isolates were assigned into 9 STs, 4 of which had only one member. The distribution of the *L. ruminis* strains and sequence types is given in Table 2. Sequence characteristics of the housekeeping gene fragments are given in Additional file 3.

**Table 2:** **Classification of** ***L. ruminis*** **strains according to sequence types**

**Host**	**Strain**	ST*	**Allele number at each locus**					
			**ftsQ**	**nrdB**	**parB**	**pheS**	**pstB**	**rpoA**
Bovine	ATCC 27782	1	1	1	1	1	1	1
Human	L5	2	2	2	2	2	2	2
	S21	2	2	2	2	2	2	2
	S36	2	2	2	2	2	2	2
	S23	3	3	3	2	3	3	3
	S38	4	3	4	3	3	4	3
	ATCC 25644	5	3	5	4	4	5	2
Bovine	ATCC 27780	6	4	6	1	5	1	4
	ATCC 27781	6	4	6	1	5	1	4
Porcine	DPC 6830	7	1	1	5	6	1	1
	DPC 6831	7	1	1	5	6	1	1
Equine	DPC 6832	8	5	7	6	7	6	5
	DPC 6834	8	5	7	6	7	6	5
	DPC 6836	8	5	7	6	7	6	5
	DPC 6833	9	6	8	6	8	7	6
	DPC 6835	9	6	8	6	8	7	6

*Sequence type,

Note: A combination of six alleles defines an allelic profile and each unique allelic profile represents a sequence type.

The bovine isolate ATCC 27782 whose genome we previously sequenced [24] was designated as ST-1 and was found to be unique in this data set. The relatively small number of isolates and loci did not allow the identification of the most prevalent ST.

**Figure 1:**
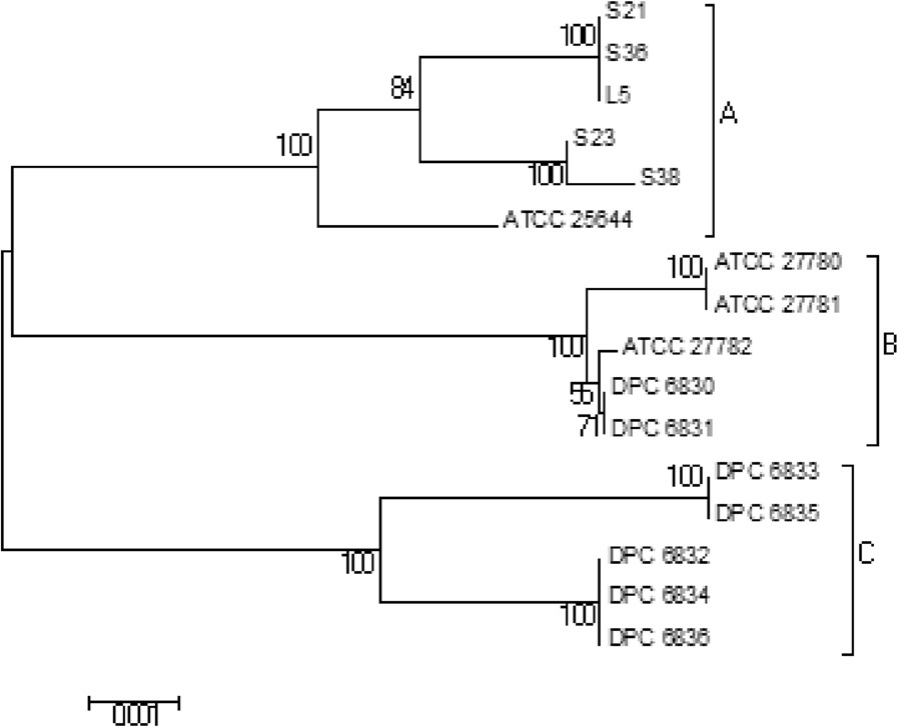
**Neighbor-joining tree based upon the concatenated sequences for six MLST loci examined in 16** ***L. ruminis*** **strains.** Three major clades are labelled **(A**-**C)**. Cluster A – strains of human origin, Cluster B – strains of bovine and porcine origin and Cluster C – strains of equine origin. Scale bar indicates 0.001 nucleotide changes.

Three clades were identified from the concatenated sequence tree of the MLST loci (Figure 1). Clade A comprised all of the human derived isolates; Clade B comprised the bovine and porcine isolates and Clade C comprised the equine isolates. Considering phylogenetic trees constructed from individual loci (Additional file 4), 4 loci (*ftsQ*, *nrdB*, *pheS*, *pstB*) produced the same 3 clades as the concatenated tree. However, *rpoA* showed 2 clades, Clade AB combining all of the human, bovine and porcine isolates into a single clade and Clade C containing the equine isolates. The *parB*-based trees showed 2 clades, with Clade BC combining all of the bovine, porcine and equine isolates. For all loci tested, the bovine and porcine strains clustered with each other indicating that they are genetically closely related.

### Comparative genomics of *L. ruminis* strains

To complement the genome sequences already available [24], four additional *L. ruminis* genomes were sequenced: strain S23, a human isolate; strain DPC 6832, a swarming equine isolate; DPC 6830, a porcine isolate, and ATCC 27780^T^, a bovine isolate and type strain. A comparison of the major genomic features of the six sequenced *L. ruminis* strains is provided in Table 3.

**Table 3:** **Major genomic features of the six sequenced** ***L. ruminis*** **strains**

**Feature**	**Host and strains**					
	**Human**		**Bovine**		**Porcine**	**Equine**
	**S23**	ATCC 25644*	**ATCC 27780**	ATCC 27782*	**DPC 6830**	**DPC 6832**
Genome size (bp)	1,932,610	2,069,085	2,046,230	2,066,647	2,067,308	1,948,988
G + C content (%)	43.7	43.7	43.3	43.4	43.3	43.0
CDS	1932	1984	1976	1976	1980	1883
Estimated Coding density (%)	85.2	85.1	84.3	84.0	83.4	83.7
tRNAs	34	49	59	67	57	58

*Information summarised from Forde *et al*. [24].

**Figure 2:**
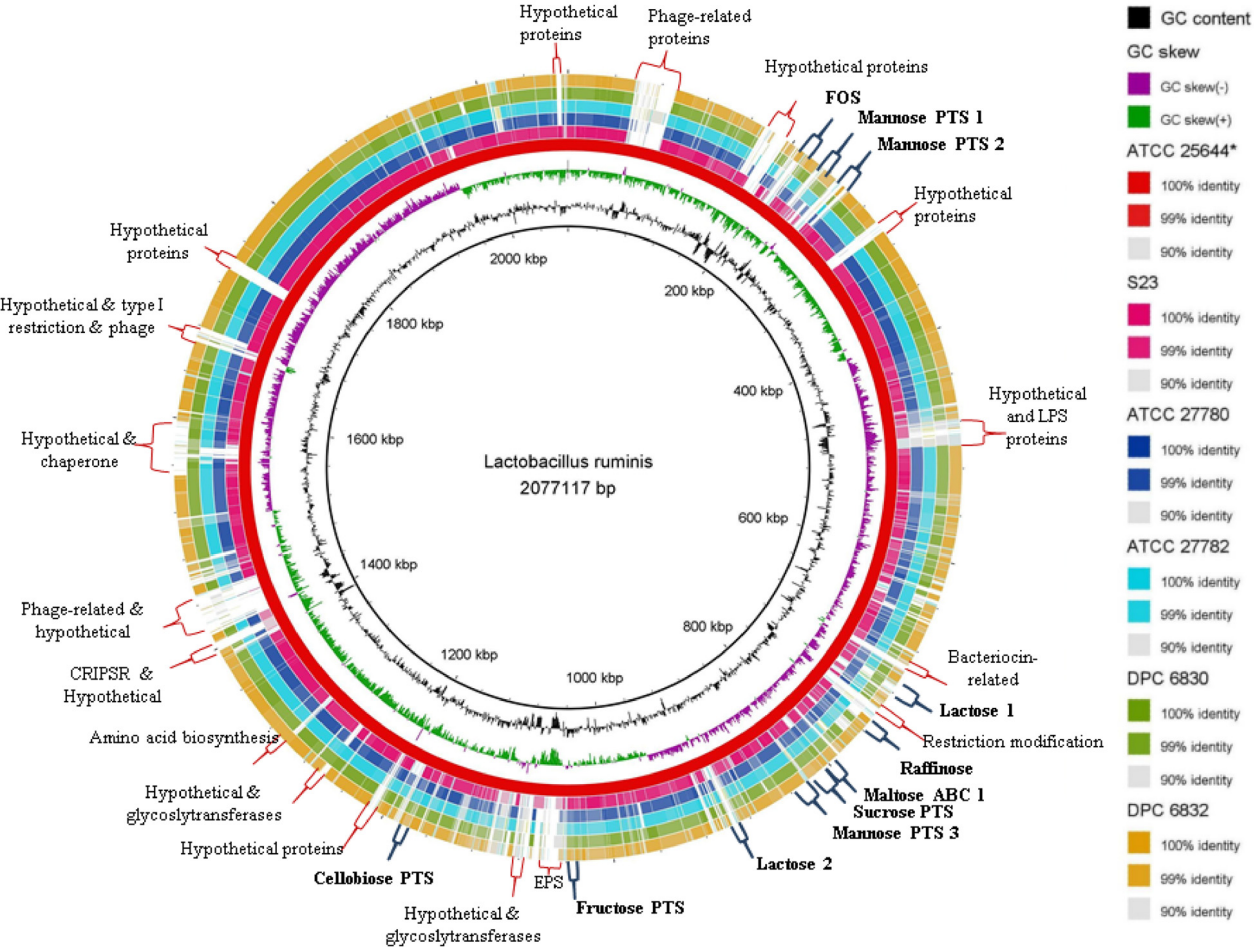
**Blast ring image generator comparison of the sequenced** ***L. ruminis*** **genomes.** The six sequenced genomes are arranged as follows: ATCC 25644 (*reference genome), S23, ATCC 22780, ATCC 27782, DPC 6830 and DPC 6832 using a 90-99% similarity threshold. Carbohydrate genes and operons are marked in bold with blue-coloured markers. Hypothetical proteins, phage-related proteins and other gaps in the sequences are marked in black.

The Blast ring image generator (BRIG) was used to graphically compare the sequenced *L. ruminis* strains to the chosen reference genome of ATCC 25644 (Figure 2). The comparison revealed large regions of similarity (99%) interspersed with small regions of dissimilarity and gaps. Examination of the BRIG image and manual curation of genomes aligned with the Artemis Comparison Tool (ACT) revealed that gaps and regions of dissimilarity in the sequence alignments were due to phage-related, hypothetical, CRISPR and restriction-modification proteins.

**Figure 3:**
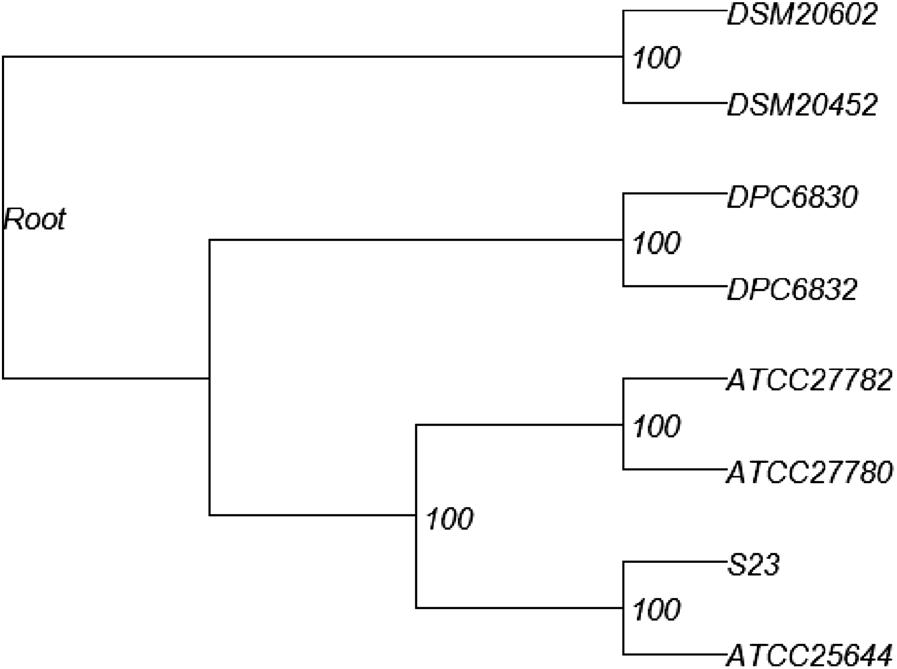
**Whole-genome phylogenetic tree for the six sequenced** ***L. ruminis*** **genomes.** The genomes of strains S23 and ATCC 25644 (human origin), ATCC 27780 and ATCC 27782 (bovine origin), DPC 6830 (porcine origin) and DPC 6832 (equine origin) were compared with two *L. salivarius* clade species as outgroups, *L. murinus* (DSM20452) and *L. animalis* (DSM20602).

To complement the carbohydrate utilisation comparison and strain relatedness analysis by MLST, we performed whole-genome phylogeny for the six *L. ruminis* genomes available. Comparisons were made using a core gene set present in the six *L. ruminis* genomes with two species from the *L. salivarius* clade as the out-group (Figure 3). Clustering of the genome sequences from the human-derived and bovine-derived strains indicate that they share a more recent common ancestor with each other than they do with the two porcine strains. From the data and the core gene tree it is clear that the two porcine-derived strains, DPC 6830 and DPC 6832, are the most divergent of the *L. ruminis* strains.

### Carbohydrate fermentation profiling

The growth profiles of the seven newly isolated *L. ruminis* strains are summarised in Table 4. None of the strains fermented ribose, consistent with the absence of the pentose phosphate pathway in *L. ruminis*. Similar to the human and bovine isolate fermentation profiles, the porcine and equine strains were able to utilise mono-, di-, tri- and tetra-saccharides. The porcine isolates were most clearly distinguished from the equine strains by the former being able to utilise lactose, lactulose and GOS inulin for growth. The porcine isolates were also able to weakly ferment sialic acid for growth. A particularly heterogeneous fermentation pattern was identified for the porcine and equine strains when grown on beta-fructofuranosides. The majority of strains were unable to ferment polysaccharides and inulins. However, DPC 6831 was able to weakly ferment cellulose. DPC 6831 and DPC 6835 were able to ferment dextran and Raftiline HP. This would suggest that that the majority of *L. ruminis* isolates are unable to ferment carbohydrates with a DP greater than 10 [23]. No demonstrable amylase activity was identified in any isolate; amylase activity is sometimes considered a desirable trait for potential probiotics.

**Table 4:** **Growth profiles for newly isolated** ***L. ruminis*** **strains on diverse carbohydrates**

**Carbohydrate class**	**Carbohydrate**	**Strain**						
		**Porcine**		**Equine**				
		**DPC 6830**	**DPC 6831**	**DPC 6832**	**DPC 6833**	**DPC 6834**	**DPC 6835**	**DPC 6836**
Mono and di-saccharides	Fructose	+	+	++	+	++	++	+
	Galactose	++	++	++	++	++	++	++
	Glucose	-	+	+++	++	++	++	++
	Lyxose	-	+	-	-	-	-	-
	Maltose	+	+	+	++	++	++	-
	Mannose	+++	+++	+++	++	++	++	+++
	Ribose	-	-	-	-	-	-	-
	Sucrose	++	+++	+++	++	++	++	++
α-galactosides	Melibiose	++	++	+++	++	++	++	++
	Raffinose	++	++	++	++	+	++	-
	Stachyose	++	++	++	++	+++	+++	+++
β-galactosides	GOS	+	+	-	-	-	-	-
	GOS Inulin	+	++	-	-	-	-	-
	Lactose	+++	++	-	-	-	-	-
	Lactulose	++	++	-	-	-	-	-
β-glucosides	β-Glucotriose B	++	++	+++	++	++	++	++
	Cellobiose	+	+	++	+	+	-	-
β-fructofuranosides & Inulins	Raftiline HP	-	+	-	-	-	+	-
	Raftiline ST	+	++	+	+	-	-	++
	Raftilose P95	-	-	++	+	+	++	++
	Raftilose Synergy 1	-	++	+++	++	++	++	++
	Dextran	-	+	-	-	-	++	-
Polysaccharides	Esculin	-	+	-	-	-	-	-
	Lichenan	-	-	-	-	-	-	-
	Sialic acid	++	+	-	-	-	+	+
	Siallylactose	+	+	ND	++	-	-	-
	Soluble Starch	-	-	-	-	-	-	-
	Cellulose	-	+	-	-	-	-	-

- = no growth, + = poor growth, ++ = moderate growth, +++ = strong growth, ND = Not done.

The carbohydrate utilization operons in four of the newly sequenced genomes were compared using ACT and the residue identities between each operon were established as shown in Additional file 5. A mannose PTS (mannose PTS1) operon present in ATCC 25644 was also identified in the genome of S23, which suggests that this operon is unique tohuman isolates. However, a second mannose PTS (mannose PTS2) operon and one of the lactose operons (*lacZ*2) [23] were only present in ATCC 25644. A high level of conservation (95-99%) both at the nucleotide and amino acid level was noted for the raffinose, glycogen, sucrose and fructose operons and the third mannose PTS operon (mannose PTS3). A fragment of the lactose operon (lacZ1) was also identified in the genome of strain S23. It consisted of the β-galactosidase enzyme and GPH transporter, but lacked the *lacI* regulator. A fragment of the maltose ABC operon was identified in the genome of strain S23. The genome of strain S23 contains only one of the two operons for lactose and maltose utilisation that are present in *L. ruminis* ATCC 25644. The fragmented operons may be a result of gaps in the draft genome of S23 as the carbohydrate fermentation profiles revealed the ability to ferment both lactose and maltose. A comparable level of similarity was observed when using the complete genome of ATCC 27782 as the reference genome in the BRIG analysis (Additional file 6).

### Biochemical and metabolic characterisation

One of the overall aims was to determine if the extended panel of *L. ruminis* strains included isolates with biochemical/metabolic traits that might allow their further development as probiotics.

Bacterial exopolysaccharides have potential uses in the food and pharmaceutical industries. The ability of 16 *L. ruminis* strains to produce EPS in the presence of three carbon sources is shown in Table 5. Forty percent of the isolates had a positive “ropy” phenotype with all of the media (glucose, sucrose and lactose) used. Four of the isolates failed to produce EPS under any conditions, but in the case of the bovine isolate, ATCC 27782, on lactose MRS plates, this was due to zero growth. Future studies will be needed to confirm these initial findings, which are of interest because of the ability of EPS production to modulate the interaction of *Lactobacillus* with the innate immune system [45].

**Table 5:** **Resistance and biochemical characteristics of** ***L. ruminis***

**Test**	**Conc./Variable**	**Human**						**Bovine**			**Porcine**		**Equine**				
		**L5**	**S21**	**S23**	**S36**	**S38**	**25644**	**27780**	**27781**	**27782**	**DPC 6830**	**DPC 6831**	**DPC 6832**	**DPC 6833**	**DPC 6834**	**DPC 6835**	**DPC 6836**
**Resistance assays**																	
Bile Salt exposure	0.25%	+	+	+	+	+	+	+	+	+	+	+	+	+	+	+	+
	0.50%	+	+	+	+	+	+	+	+	+	+	+	+	+	+	+	+
	0.75%	-	+	+	+	+	+	+	+	+	+	+	+	+	+	+	+
	1%	-	+	+	+	+	+	+	+	+	+	+	+	+	+	-	+
	2%	-	-	-	+	+	+	-	-	-	-	+	+	+	+	-	-
	5%	-	-	-	-	+	-	-	-	-	-	-	+	-	-	-	-
pH resistance	5.5	+	+	+	+	+	+	+	+	+	+	+	+	+	+	+	+
	4.5	-	-	-	-	-	-	-	+	+	+	+	+	+	+	+	+
	4	-	-	-	-	-	-	-	+	-	+	+	+	+	+	+	+
	3.5	-	-	-	-	-	-	-	-	-	-	+	+	+	+	+	+
	3	-	-	-	-	-	-	-	-	-	-	-	+	+	+	+	+
Chloramphenicol sensitivity	≥4 μg/ml	S	S	S	S	R	R	S	S	R	R	S	S	R	R	S	R
Rifampicin sensitivity	≥1 μg/ml	S	S	S	S	S	S	S	S	S	S	S	S	S	S	S	S
Simulated gastric juice	Survival (%)	54	53	86	36	60	63	44	50	45	66	67	78	85	82	71	83
**Biochemical assays**																	
OPNG (β-galactosidase)		+	+	+	+	+	+	+	+	-	+	+	-	-	-	-	-
API-ZYM	Leucine arylamidase (EC. 3.4.11.1)	++	++	++	+	++	++	++	++	++	++	++	++	++	++	++	++
	Valine arylamidase	++	++	++	+	++	++	++	++	+	++	++	+/−	+	++	++	++
	Cystine arylamidase (EC. 3.4.11.3)	+	+/−	+/−	-	+/−	+	+	+/−	-	-	+/−	-	-	-	+/−	-
	Acid phosphatase (EC. 3.1.3.2)	+/−	+/−	+	+/−	+/−	+/−	++	+	+/−	+	+	++	+	+	+/−	++
	Naphthol-AS-BI-phosphohydrolase	+	+	+	+	+/−	+/−	+	+	+	+	+	+	+	+	+	+
	α-galactosidase (EC. 3.2.1.22)	+	+	+	+/−	+/−	+	+	+	+	++	++	++	+	+/−	+/−	+
	β-galactosidase (EC. 3.2.1.23)	++	++	++	++	++	++	++	++	-	++	++	-	-	-	-	-
	β-glucuronidase (EC. 3.2.1.31)	+/−	-	-	+/−	-	+/−	-	+/−	+/−	-	+/−	-	-	-	-	-
	α-glucosidase (EC. 3.2.1.20)	+/−	-	-	-	-	+	+/−	+/−	+	++	+	+	-	-	-	+
	β-glucosidase (EC. 3.2.1.21)	+/−	+	+	+/−	+/−	+	-	-	+	-	-	-	+/−	-	-	-
	N-acetyl-β-glucosaminidase (EC. 3.2.1.52)	-	-	-	-	-	-	-	-	-	-	-	-	-	-	-	-
	α-mannosidase (EC. 3.2.1.24)	-	-	-	-	-	-	-	-	-	-	-	-	-	-	-	-
	α-fucosidase (EC. 3.2.1.51)	-	-	-	-	-	-	-	-	-	-	-	-	-	-	-	-
	α-chymotrypsin (EC. 3.4.21.1)	-	-	-	-	-	-	-	-	-	-	-	-	-	-	-	-
	Trypsin (EC. 3.4.21.4)	-	-	-	-	-	-	-	-	-	-	-	-	-	-	-	-
	Alkaline phosphatase (EC. 3.1.3.1)	-	-	-	-	-	-	-	-	-	-	-	-	-	-	-	-
	Esterase (C4)	-	-	-	-	-	-	-	-	-	-	-	-	-	-	-	-
	Esterase lipase (C8)	-	-	-	-	-	-	-	-	-	-	-	-	-	-	-	-
	Lipase (C14)	-	-	-	-	-	-	-	-	-	-	-	-	-	-	-	-
EPS production	Glucose-MRS	+	+	-	+	-	+	+	+	-	+	-	+	-	+	-	+
	Sucrose-MRS	+	+	-	+	-	-	+	+	-	+	+	+	-	+	+	-
	Lactose-MRS	+	+	-	+	-	-	+	+	-	+	+	-	-	-	-	-

++ strongly positive; + positive; − negative; +/− weak; S susceptible; R resistant.

All strains were susceptible to the broad-spectrum antibiotic rifampicin (Table 5). Seven strains were resistant to 4 μg/ml of chloramphenicol. The resistant strains included the two strains best characterized to date, ATCC 25644 and ATCC 27782, which may not therefore be suitable for further investigation as probiotic strains.

**Table 6:** **Genes differentially expressed in** ***Lactobacillus ruminis*** **ATCC 27782**

**Primer pair**	**ATCC 27782**								**Function**
	**ID**	**RNA-seq**					**RT-PCR**		
		**Swimming vs. Stationary log2 fold change** ^**a**^	**pval**	**Swarming vs. Stationary log2 fold change** ^**b**^	**pval**	**Fold change**	**2**ΔΔ**CT fold change swimming vs. Stationary** ^**c**^	**2**ΔΔ**CT fold change swarming vs. Stationary** ^**d**^	
MMOD 1	LRC_00640	−2.97	*	−0.03	>0.05	8.00			hypothetical protein
MMOD 2	LRC_00780	−5.54	***	−0.94	>0.05	24.00	0.02 (0.02-0.02)	7.09 (6.35-7.93)	DeoR family transcriptional regulator
MMOD 3	pfkB	−4.10	**	−1.54	>0.05	6.00	0.01 (0.01-0.01)	0.08 (0.06-0.10)	1-phosphofructokinase
MMOD 4	LRC_00800	−3.18	*	−0.74	>0.05	5.00	0.2 (0.19-0.21)	0.19 (0.16-0.23)	PTS system fructose-specific
MMOD 5	LRC_03250	2.62	>0.05	−0.60	>0.05	9.00	17.92 (17.39-18.46)	0.22 (0.18-0.27)	hypothetical protein
MMOD 6	LRC_04370	0.96	>0.05	1.00	>0.05	1.03	1.06 (1.01-1.10)	0.22 (0.18-0.26)	hypothetical_protein
MMOD 9	LRC_05780	4.04	**	0.89	>0.05	9.00	28.54 (28.01-29.08)	0.27 (0.23-0.32)	hypothetical protein
MMOD 10	LRC_06170	0.83	>0.05	−1.16	>0.05	3.99	0.74 (0.70-0.77)	0.41 (0.33-0.50)	flagellin
MMOD 11	iD = LRC_04600	1.35	>0.05	2.42	>0.05	2.10	8.31 (7.94-8.70)	0.62 (0.51-0.75)	hypothetical_protein
MMOD 12	fliC	1.27	>0.05	0.16	>0.05	2.15	1.83 (1.83-1.83)	0.44 (0.36-0.54)	flagellin
MMOD 13	LRC_15700	1.07	>0.05	0.20	>0.05	1.82	1.48 (1.41-1.55)	0.31 (0.25-0.38)	flagellin
MMOD 14	LRC_18780	5.11	**	−0.48	>0.05	48.00	94.03 (89.54-98.74)	0.19 (0.16-0.23)	PTS system sucrose-specific transporter subunit IIABC
MMOD 15	LRC_16260	3.70	*	1.16	>0.05	6.00	2.13 (2.06-2.21)	0.05 (0.04-0.07)	hypothetical protein

a – negative values indicate a down-regulation in the swimming cells.

b – negative values indicate a down-regulation in the swarming cells.

c – values below 1 indicate a down-regulation of swimming cells.

d – values below 1 indicate a down-regulation of swarming cells.

Note: * = P ≤ 0.05, ** =P ≤ 0.01, *** = P ≤ 0.001.

Characterisation of enzymatic activity is an important tool for validating genome annotations. API-ZYM was used as a semi-quantitative method to identify enzymatic activity in the 16 *L. ruminis* isolates (Table 5). All of the strains tested were positive for leucine arylamidase, valine arylamidase, α-galactosidase, Napthol-AS-BI-phosphohydrolase, N-acetyl-β-glucoaminidase and acid phosphatase. β-glucosidase activity was identified in all of the human isolate strains, in DPC 6833 (equine) and ATCC 27782 (bovine). Weak β-glucuronidase activity was noted in some of the strains tested (L5, S36, ATCC 27781 and DPC 6831). No activity was detected for the majority of strains for alkaline phosphatase, esterase (C4), esterase lipase (C8), lipase, trypsin, α-chymotrypsin, α-mannosidase or α-fucosidase. ONPG discs detected the production of β-galactosidase in all of the human and porcine isolates and two bovine isolates (ATCC 27780 and ATCC 27781). No activity was seen for ATCC 27782 or any of the equine isolates. This concurs with the carbohydrate fermentation profiling and the API-ZYM assays.

### Resistance profiling and stress resistance

All of the strains were able to grow in porcine bile salts at a concentration of ≤0.5% (w/v) as shown in Table 5. The equine and porcine isolates displayed the highest resistance to bile. None of the human isolates were able to grow in MRS with a pH below 5.5. This may indicate that these strains would be unable to survive the pH stress of gastric transit. Only the equine strains were able to tolerate the lowest pH values tested (3.5-3.0). The simulated gastric juice (SGJ) survival assay combined the bactericidal effects of low pH and digestive enzymes. Variable strain-dependent reductions in cell numbers occurred after 3 hours treatment (Table 5 and Additional file 7). After 24 hours, all of the strains showed a complete loss of viability (data not shown). Strains S23, DPC 6833 and DPC 6836 showed the highest survival rate in SGJ with just over a 1 log reduction in cell numbers after 3 hours. Strains L5, S21, S36 and ATCC 27780 were the most sensitive to SGJ with a 4–5 log reduction in cell numbers after 3 hours. All the reductions were statistically significant (p < 0.05).

Technological stresses are commonly applied to microbes in functional foods. The ability to tolerate technological stresses like an oxygen-rich environment and high saline conditions are therefore important first-stage characteristics to examine when screening a culture bank for strains of potential use and further testing as candidate probiotics. All of the *L. ruminis* strains were able to grow in medium supplemented with up to 3% NaCl (Additional file 8). With the exception of the equine strains, DPC 6835 and DPC 6836, concentrations of NaCl above 4% were inhibitory to growth. The ability of a strain to grow in milk is a benefit for use in a dairy-based delivery vector. Milk acidifying capacity was examined by growing each strain in milk over a 72-hour period and comparing each culture to the negative control (pH 6.3). DPC 6834 was unable to grow and acidify milk. All of the other strains tested were able to ferment milk, producing final pH values ranging from pH 4.1 to pH 5.1. Future studies will be needed to assess the organoleptic characteristics of the *L. ruminis*-fermented milk.

Bacterial oxygen tolerance is an advantageous trait, which simplifies culturing and processing. The growth of the majority of the human and bovine isolates was reduced by oxygen exposure, with a median 81% reduction in final culture absorbance for the human isolates and 73% of a reduction for the bovine strains. A reduction in final absorbance values of 4% and13% was noted for the porcine and equine strains, respectively. Thus, the porcine and equine *L. ruminis* strains were aero-tolerant and as such are suitable candidates for probiotic processing.

### Assessment of motility

**Figure 4:**
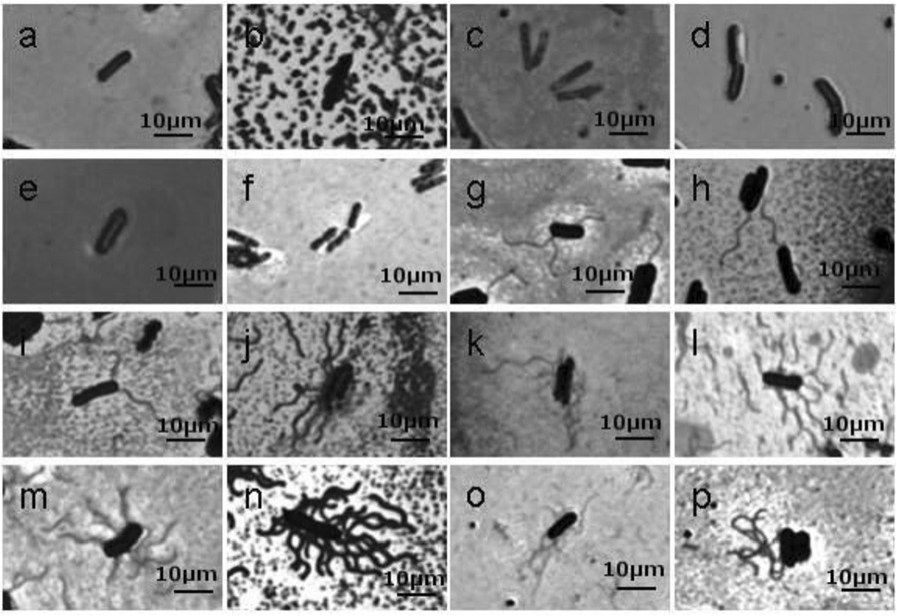
**Flagellum staining of 16 strains of** ***Lactobacillus ruminis*** **using light microscopy. (a)** L5, **(b)** S21, **(c)** S23, **(d)** S36, **(e)** S38, **(f)** ATCC 25644, **(g)** ATCC 27780, **(h)** ATCC 27781, **(i)** ATCC 27782, **(j)** DPC 6830, **(k)** DPC 6831, **(l)** DPC 6832, **(m)** DPC 6833, **(n)** DPC 6834, **(o)** DPC 6835, **(p)** DPC 6836.

Motility of *L. ruminis* is also a strain-variable trait [19]. Flagellum staining of cells of the sixteen strains confirmed our previous observation that all of the human isolates were not motile, since they lacked any visible flagella or remnants of flagella (Figure 4). In contrast, all of the bovine, porcine and equine strains produced flagella. The bovine strains had between 1–2 flagella attached per cell. The porcine and equine strains had between 4 and 16 peritrichous flagella per cell, with an average number of 6 flagella per cell.

Swarming is a form of motility on a solid surface. On lower concentrations of agar (≤0.5% w/v) all of the strains tested (of bovine, porcine and equine origin) had the ability to swarm (data not shown). None of the human strains had the ability to swarm. The bovine strains, ATCC 27780, ATCC 27781 and ATCC 27782 were only able to swarm on MRS plates with 0.5% (w/v) agar, which classifies them as soft swarmers [46]. Additional file 9 shows representative data from the swarm assays for porcine and equine strains, which were able to swarm on MRS plates with an agar concentration up to 1.8% (w/v). These strains are therefore classified as hard swarmers [46]. The presence of increasing concentrations of the biosurfactant Tween 80 did not enhance swarming. All strains (excluding human-derived) were able to swarm at the lowest concentration of Tween 80 (0.1% v/v) present in MRS media as standard. Altering the carbohydrate and reducing the concentration from 2 to 0.5% (w/v) negatively impacted ability to swarm. The swarming phenotype was absent in the porcine and equine strains when grown on any carbohydrate other than glucose at a reduced concentration of 0.5% (w/v). Therefore, carbohydrate and agar concentrations are key factors in the ability of a strain to swarm.

### Transcriptome analysis of *L. ruminis* motility

In this study, we applied RNA sequencing (RNA-seq) as a high-throughput screening method to provisionally identify differentially expressed swimming- or swarming-associated genes. The expression of these candidate genes was then further examined by qRT-PCR. The ATCC 27782 and DPC 6832 strains grown on agar plates containing 0.5% agar (swarming), 2% agar (stationary) and MRS broth (swimming) constituted the six samples analysed. The total number of aligned sequences for *L. ruminis* ATCC 27782 and DPC 6832 was 30,227,006 and 30,577,156, respectively. One hundred and nine genes and 19 genes were identified as being differentially expressed (p < 0.05) from the RNA-seq data in *L. ruminis* ATCC 27782 swimming and swarming cells, respectively, when compared to the stationary growth control. Eighty-nine genes (DPC swimming) and 30 genes (DPC swarming) were identified as being differentially expressed (p < 0.05) from the RNA-seq data in *L. ruminis* DPC 6832 swimming and swarming, respectively, when compared to the stationary growth control. These differentially expressed genes are shown in Additional file 10 and Additional file 11 for *L. ruminis* ATCC 27782 and DPC 6832, respectively. From the RNA-seq data, we identified 15 genes for further studies, where functions were divided into flagellar biosynthesis, carbohydrate utilisation and hypothetical proteins. These 15 genes were examined by qRT-PCR to quantify and confirm the level of differential expression in swarming or swimming cells compared to stationary cells. The data generated from the 15 genes for both RNA-seq and RT-PCR is shown in Tables 6 and 7 for ATCC 27782 and DPC 6832, respectively. The majority of the differentially expressed genes identified in *L. ruminis* ATCC 27782 were identified as ribosomal proteins (Additional file 10) that are essential for growth and proliferation of cells in general and were therefore excluded from further analysis.

**Table 7:** **Genes differentially expressed in** ***Lactobacillus ruminis*** **DPC 6832**

**Primer pair**	**DPC 6832**								**Function**
	**ID**	**RNA-seq**					**RT-PCR**		
		**Swimming vs. Stationary log2 fold change** ^**a**^	**pval**	**Swarming vs. Stationary log2 fold change** ^**b**^	**pval**	**Fold change**	**2**ΔΔ**CT fold change swimming vs. Stationary** ^**c**^	**2**ΔΔ**CT fold change swarming vs. Stationary** ^**d**^	
MMOD 1	LRN_87	−3.36	**	1.81	>0.05	36	12.92 (9.72-17.18)	105.18 (95.45-115.89)	hypothetical protein
MMOD 2	LRN_108	−5.11	***	2.66	*	218	2.80 (2.40-3.24)	4.34 (3.21-5.87)	DeoR family transcriptional regulator
MMOD 3	LRN_109	−3.94	**	3.62	**	189	0.06 (0.06-0.07	7.80 (6.01-10.13)	1-phosphofructokinase
MMOD 4	LRN_110	−2.93	*	4.29	**	149	0.02 (0.02-0.03)	6.06 (4.40-8.35)	PTS system fructose-specific
MMOD 5	LRN_324	2.50	*	−0.86	>0.05	10	0.97 (0.74-1.27)	0.11 (0.08-0.14)	hypothetical protein
MMOD 6	LRN_409	−4.62	***	2.28	>0.05	120	0.03 (0.02-0.04)	1.03 (0.81-1.29)	hypothetical_protein
MMOD 7	LRN_520	−0.74	>0.05	3.39	**	18	0.49 (0.36-0.68)	7.35 (5.57-9.71)	beta-fructofuranosidase
MMOD 8	LRN_521	0.06	>0.05	4.49	**	22	0.16 (0.12-0.21)	6.07 (4.61-7.98)	MFS Transporter Beta fructofuranosidase
MMOD 9	LRN_561	1.83	>0.05	−1.15	>0.05	8	0.58 (0.43-0.77)	0.12 (0.09-0.15)	hypothetical protein
MMOD 10	LRN_598	1.24	>0.05	0.67	>0.05	1.48	0.24 (0.18-0.33)	0.46 (0.35-0.62)	flagellin
MMOD 11	LRN_933	5.15	***	3.24	*	4	1.42 (1.20-1.70)	0.72 (0.53-0.98)	hypothetical_protein
MMOD 12	LRN_1405/1777	1.70	>0.05	2.89	*	2.28	0.46 (0.33-0.62)	2.09 (1.54-2.84)	flagellin
MMOD 13	LRN_1410	1.81	>0.05	3.04	*	2.36	0.3 (0.27-0.32)	1.78 (1.31-2.42)	flagellin
MMOD 14	LRN_1655	0.94	>0.05	1.84	>0.05	1.87	0.23 (0.18-0.29)	0.27 (0.20-0.36)	PTS system sucrose-specific transporter subunit IIABC

a – negative values indicate a down-regulation in the swimming cells.

b – negative values indicate a down-regulation in the swarming cells.

c – values below 1 indicate a down-regulation of swimming cells.

d – values below 1 indicate a down-regulation of swarming cells.

Note: * = P ≤ 0.05, ** =P ≤ 0.01, *** = P ≤ 0.001.

No statistically significant alteration in gene expression was identified in the flagellar locus of ATCC 27782 by RNA-seq comparing stationary and motile cells. This strain does not swarm. However, nineteen flagellar locus genes were significantly differentially expressed in DPC 6832 comparing these conditions (Additional file 11). Of particular interest were the two gene copies encoding flagellin that were up-regulated in both the swimming and swarming cells in this strain. The two flagellin genes were also up-regulated in the swimming cells of ATCC 27782 in the RNA-seq dataset, but did not reach statistical significance (p > 0.05). Examination of the differentially expressed genes reaching statistical significance in both strains revealed that the fructose utilisation operon (LRN_108-110) was significantly down-regulated in swimming and swarming cells in ATCC 27782 and significantly up-regulated in the swarming cells of DPC 6832 in the RNA-seq dataset. However, examination of the qRT-PCR data showed that the DeoR fructose transcriptional regulator (LRN_108 and LRC_00780) was also up-regulated in swarming ATCC 27782 cells and in the swimming DPC 6832 cells. Other carbohydrate metabolism genes were significantly differentially expressed in both strains. The sucrose PTS transporter (LRC_18780) was significantly up-regulated in motile ATCC 27782 cells, in both swarming and swimming cells compared to stationary. This suggests that this transporter plays an unknown role in motility in *L. ruminis* ATCC 27782. Two genes that form part of the fructooligosaccharide utilisation operon (LRN_520-521) were up-regulated in swarming cells in DPC 6832 (in both the RNA-seq and RT-PCR datasets). Genes for a number of hypothetical proteins were also significantly up- or down-regulated in the RNA-seq dataset, including the hypothetical proteins LRC_03250/LRN_324 and LRC_05780/LRN_561. However, in DPC 6832 only LRN_324 was up-regulated in both the RNA-seq and the qRT-PCR datasets. The hypothetical protein LRN_87 may be important for swimming and swarming cells in DPC 6832 based upon a significant up-regulation of this gene in the qRT-PCR analysis. Hypothetical proteins unique to a particular strain may also play a part in swimming or swarming in their respective strains; for example LRC_16260 may be essential for motility in ATCC 27782. This hypothetical gene was up-regulated in swimming cells in both datasets. However, while these hypothetical proteins appear to be important for swimming and swarming motility in *L. ruminis,* functional investigation is needed to verify their importance in the different motility phenotypes.

## Discussion

*Lactobacillus ruminis* is a member of the mammalian gut microbiome and is regarded as an autochthonous species in humans [3]. In this study, we aimed to determine the genomic diversity and biochemical and metabolic characteristics of the known *L. ruminis* isolates. To date, *L. ruminis* has only been isolated and identified in the lower intestines and has therefore been overlooked as a potential probiotic with the ability to survive upper intestinal tract conditions. A battery of tests were carried out to simulate the conditions faced by a strain as it migrates through the gastrointestinal tract [26]. In this study, 63% of the strains (n = 10) showed an ability to survive the simulated gastric juice (at greater than 60% of their original population numbers) *in vitro*. The survival rates for *L. ruminis* in SGJ (36-85%) were similar to those of human isolates of *L. plantarum* [47]. These data indicate that *L. ruminis* has the potential to survive gastric transit at tolerance levels comparable to robust lactobacilli. All of the strains showed resistance and the ability to grow in media containing up to 0.75% (w/v) bile salts. This is higher than the levels estimated to be found in the intestines [26]. Testing with increasing concentrations of bile salts, higher than those found *in vivo*, revealed that 44% of the isolates were able to grow in the presence of up to 2% bile salts. This is consistent with similar tests carried out on other human-derived *Lactobacillus* spp. including *L. ruminis* isolated from the faecal samples of healthy Spanish volunteers [48-50]. Low pH was identified as a major growth-limiting factor for *L. ruminis* strains with less than half of the isolates tested able to survive a pH of 4.5. Similar levels of survival at pH 4.5 were noted by Delgado and colleagues using other *L. ruminis* strains [49]. The data generated here and by Delgado *et al.* [49] suggest that *L. ruminis* has a high tolerance to bile salts and that human-derived strains are susceptible to acidic pH. However, all equine isolate strains tested here were able to survive and maintain minimal growth at pH 3.0. This suggests that the equine strains have evolved a greater tolerance to low pH and this was also reflected in the response of these strains to SGJ.

Antibiotic resistance is a global problem for healthcare providers and for human and animal health. The possibility of horizontal transfer of resistance genes *in vivo* means it is important to assess a strain’s resistance to a variety of antibiotics [51]. Due to the high level of aminoglycoside antibiotic resistance that is endogenous to the *Lactobacillus* species [52] and some initial tests carried out in this study (data not shown), these antiobiotics were omitted. Instead, we focused on the broad-spectrum antibiotics chloramphenicol and rifampicin. Using the EFSA guidelines [53], 44% of the *L. ruminis* strains were de-selected from the probiotic assessment based on their resistance of up to 4 ug/ml of chloramphenicol. However, due to noted resistance and safety concerns, chloramphenicol is no longer used as a common antibiotic in medicine [54] and these resistant strains may be revisited in the future for further probiotic assessment. All of the isolates were susceptible to rifampicin.

The catabolic flexibility of mammalian-derived lactobacilli is important for their survival in the gastrointestinal tract [55]. Assessment of the prebiotic utilisation of each individual strain has the potential to allow for the creation of targeted synbiotic products. The ability of each *L. ruminis* strain to ferment at least one class of prebiotic carbohydrate is indicative of its adaptation to the lower gastrointestinal tract rich in NDO. The combination of a prebiotic with an *L. ruminis* culture could be used to modulate the microbiota of humans and animals. However, further testing would be required to assess the efficacy of a synbiotic treatment on the microbiota. The additional copies of the lactose and maltose operons identified in the genome of ATCC 25644 when compared to the other human-derived strain, S23, are indicative of horizontal transfer from another species present in the human microbiome.

Technological assessment of the potential probiotics was assessed by monitoring growth in a high saline environment. All strains were able to grow in up to 3% NaCl and the majority of the isolates were able to tolerate and grow in 4% NaCl. No growth was identified for any isolate in media supplemented with 6% NaCl, indicating that NaCl concentrations between 4% and 6% exert an inhibitory effect on the *L. ruminis* strains. The ability to grow and survive in an aerobic environment is also a positive technological attribute for a potential probiotic. Aerobic conditions negatively impacted the growth of the majority of human and bovine isolates. The porcine and equine isolates showed very little inhibition in their growth when exposed to the aerobic environment. The ability to survive in aerobic and saline environments suggests that the equine isolates of *L. ruminis* can be further investigated as probiotic candidates.

The β-galactosidase activity of potential probiotics may be a positive attribute in individuals suffering from lactose intolerance [56]. Six isolates expressed no β-galactosidase activity but the remaining isolates (n = 10) were able to ferment beta-galactosides. The presence of beta-galactosidase enzymatic activity in the human, bovine and porcine strains is most likely a niche adaptation. Humans, steers and weanlings (from which both porcine strains were identified) are more likely to have consumed milk and other lactose products. An exception to this was ATCC 27782, a bovine isolate strain, which lacks the ability to utilise lactose. The horses used in this study were mature racehorses and had not received any lactose-related feed in recent years. All of the equine isolates were unable to utilise lactose.

The MLST scheme described here showed high discriminatory power since it was able to differentiate between highly similar isolates. Unlike other MLST schemes [57] and studies, we found an association between sequence types, clades and the isolation source of each strain. The clade groupings identified by MLST were different from those identified by sequencing the 16S rRNA gene (additional file 2). This highlights the need to use a multi-testing (polyphasic) approach for the identification of strains and species. The efficacy of MLST for the phylogenetic comparison of bacterial genomes is based on the fact that the variability of housekeeping genes is largely unaffected by selection pressures [57]. The dN/dS ratio for each locus were less than 1, which indicates that they are not subject to positive selection and have neutral variability and were therefore suitable for use in the MLST scheme. Comparing the housekeeping gene nucleotide diversity values to other lactobacilli [57,58] revealed a higher level of polymorphism in the housekeeping genes examined in *L. ruminis*. This higher value may be related to different housekeeping genes used by the MLST scheme to generate the nucleotide diversity estimates. The application of this MLST analysis scheme on larger numbers of *L. ruminis* isolates could improve our knowledge of *L. ruminis* population structure.

Some of the phenotypic analyses corroborated the groupings identified in the MLST scheme. The equine isolates and human isolates clustered together when analysed for their tolerance of bile salts, pH stress, salt and gastric juice. This highlights the weakness of relying on phenotypic diversity alone to differentiate between strains and species. Similar phenomena were noted in *Lactobacillus delbrueckii* subspecies when grown in media supplemented with lactose [59].

N-acetyl-β-glucosaminidase production was not detected in the API-ZYM assay but the corresponding gene was present in all the genome sequences. Despite half of strains in the API-ZYM test lacking β-glucosidase activity, all of the strains were able to ferment β-glucosides *in vitro*. Both leucine and cysteine arylamidase activity was identified in each isolate. However, examination of the available *L. ruminis* genome sequences (S23, ATCC 25644, ATCC 27782 and DPC 6832) failed to identify any enzymes consistent with either leucine arylamidase or cysteine arylamidase. False negative and positive results identified using the API-ZYM assay reflect the problem in using chromogenic assays alone for assessing the presence of enzymes in bacteria.

Swarming is a type of flagellum-mediated translocation in the presence of an extracellular slime matrix. This slime matrix has been identified in many Gram negative species and is often composed of bio-surfactants, carbohydrates and proteins [60]. The increased number of hyper-flagellate, elongated cells noted in this study may also be a factor in the *L. ruminis* strains’ ability to swarm on higher concentrations of agar (1–1.8%). To elucidate the genes transcribed during swarming and swimming in *L. ruminis* a combination of molecular and high-throughput sequencing techniques were used. RNA sequencing has previously been used to study the swimming motility in *L. ruminis* L5 in response to a medium supplemented with cellobiose [37]. In the present study we focused on the motility of cells grown in un-supplemented MRS media. We identified 14 genes in both motile *L. ruminis* strains, ATCC 27782 and DPC 6832, which were differentially expressed between the two motility phenotypes. Unlike other studies where flagellar locus genes were significantly up-regulated when examining swimming motility [19,37,42], few flagellar locus-associated genes were significantly up-regulated here.

Swarming assays in the pathogen *Salmonella* have revealed that swarming cells have a different metabolism compared to swimming cells grown in the same nutrient medium [61]. This difference in metabolism is reflected in the use of metabolic pathways in novel ways. Kim & Surette identified an up-regulation in expression of flagellin when comparing swimming and swarming *Salmonella Typhimurium* cells [61]; a similar up-regulation in flagellin gene expression for both motile phenotypes was identified in the current study. Furthermore, the expression of a number of carbohydrate metabolism and transport genes was significantly up-regulated. This suggests that carbohydrate metabolic components, especially PTS transporters, play a heretofore unrecognised role in swimming and, more specifically, swarming in *Lactobacillus ruminis*, perhaps for generating extracellular slime to promote swarming. Studies in other bacteria have noted a relationship between chemotaxis and the phosphotransferase transport system [62]. However, in these studies the swimming or swarming response was restricted to the PTS-specific carbohydrate present in the test medium [62]. In the current study, glucose was present as a carbon source for each motility-related condition, but nevertheless there was an up-regulation in expression of genes related to fructose, FOS and sucrose metabolism. Further characterisation studies are needed to identify the role of the carbohydrate metabolism genes and transporters in the motile phenotypes of *L. ruminis*.

The expression of a number of hypothetical proteins was also up-regulated in the motile cells. It is possible that these hypothetical proteins may contribute to some form of novel glycolipid or lipo-peptide which acts as a bio-surfactant, facilitating swarm proliferation. However, until further characterisation work is carried out, it is impossible to say what function these proteins have in the motile phenotypes. The data generated here on the differences between swimming and swarming cells suggest that swarming cells are a distinct cell type with novel pathways that need to be investigated further.

## Conclusions

*L. ruminis* strains S23, DPC 6832 and DPC 6835 were identified as the best candidates for further testing and potential use in the future as probiotics. This is based on their ability to survive gastric and industrial stresses, and their lack of antibiotic resistance genes. The MLST scheme designed and used in this study was sufficient to distinguish isolates and their original hosts. *In vitro* analysis of *L. ruminis* showed that agar concentration, carbohydrate type, carbohydrate concentration and hydration of the agar surface are important factors in swarming phenotypic development. The transcriptional studies identified carbohydrate metabolism as an important factor for swarming cells in both motile *L. ruminis* strains. This behaviour differs from that observed for swimming cells and suggests that swarming cells may have evolved novel metabolic pathways to facilitate agar surface translocation. However, further studies are needed to elucidate the function of these metabolic genes and pathways in motile *L. ruminis* cells.

## Methods

### Bacterial strains, media and culture conditions

Bacterial strains used in this study are listed in Table 1, which includes six previously examined strains that had been isolated from human faeces and three strains isolated from cows [23]. All strains were stored at −80°C in de Man-Rogosa-Sharpe (MRS) broth (Difco BD, Ireland), supplemented with 25% (v/v) glycerol as a cryoprotectant. *Lactobacillus* strains were grown anaerobically on MRS agar plates at 37°C for two days. Growth tests (stress resistance, aerotolerance, etc.) were initiated by growing *Lactobacillus* strains anaerobically in MRS broth at 37°C overnight [23]. When required, MRS medium [63] was modified by the omission of dextrose and the addition of 0.5% (w/v) raffinose. MRS and Raffinose-MRS were used as plating media for the isolation of *L. ruminis* from porcine and equine faecal matter.

Carbohydrate-free MRS (cfMRS) [23] with added bromocresol purple was used as a basal screening medium to study the ability of the potential *Lactobacillus ruminis* strains to utilise various carbohydrates. These carbohydrates were used as a selective method to isolate *L. ruminis* based on its carbohydrate fermentation profile [23]. The carbohydrate-free MRS was supplemented with 0.5% (v/v) of cellobiose, Raftilose P95 (Beneo-Orafti, Belgium), mannitol or ribose for screening the porcine faecal isolates while the additional carbohydrates glucose, lactose, raffinose, Raftiline HP (Beneo-Orafti, Belgium) and sucrose were used in the screening of the equine faecal isolates. Mannitol and ribose were used as negative controls, i.e. carbohydrates that *L. ruminis* is unable to metabolise. All carbohydrates were provided by Sigma Aldrich, Ireland unless stated otherwise.

To characterise the swarming phenotype of *L. ruminis* isolates, MRS medium was modified in several ways: (i) addition of increasing concentration of agar from 0.5% up to 3%; (ii) addition of increasing concentrations of Tween 80 from 0.2% up to 1%; (iii) minimal MRS containing 0.5% (w/v) of four different carbohydrates – glucose, lactose, cellobiose and Raftilose P95.

### Animals and diets

Faecal samples were collected from four Large White x Landrace cross weanlings and sows. The animals were housed in the pig production unit of Teagasc Moorepark, Fermoy, Ireland. The weanlings were 10–12 weeks old. Their diets consisted of barley, wheat, maize, full-fat soya, soya hi-pro, fat, amino acids, vitamins and minerals.

Faecal samples were also collected from six mature racehorses, which were housed in a stable in Co. Limerick, Ireland. The horses were fed on diets containing forage and a high-starch concentrate [64]. All samples were collected in accordance with current Irish legislation on animal handling.

### Simulated gastric juice

To simulate the gastric environment, a sterile electrolyte solution [65] containing NaCl 6.2 gL^−1^, KCl 2.2 gL^−1^, CaCl_2_ 0.22 gL^−1^ and NaHCO_3_ 1.2 gL^−1^ was supplemented with lysozyme and pepsin (Sigma-Aldrich, Wicklow, Ireland) to final concentrations of 0.01% and 0.3% (w/v), respectively. The pH of the solution was reduced to pH 2.0 using 1 M HCl. Five millilitre volumes of each overnight culture were centrifuged at 4,000 × g for 10 min. The cell pellets were then re-suspended in the simulated gastric juice (SGJ) and incubated for 24 hours. Viable counts were determined by plate culture after 0-hr, 3-hr and 24-hr incubation.

### Carbohydrate fermentation profiling

Newly isolated porcine and equine *L. ruminis* strains were tested for their ability to utilise twenty-eight carbohydrates and compared to previously determined carbohydrate utilisation profiles for 9 other *L. ruminis* strains [23]. Each carbohydrate was filter-sterilised into cfMRS at a concentration of 0.5% (w/v). A Synergy 2 plate reader (BioTek Instruments Inc., Vermont, US) with Gen5 software was used to measure absorbance at the beginning (0 hr) and a second reading was taken after 48 hr. The carbohydrates tested include cellulose, dextran, esculin, lichenan, lyxose, Raftiline HP (Beneo-Orafti, Belgium), Raftiline ST (Beneo-Orafti, Belgium), ribose, sialic acid, sialyllactose, soluble starch, trehalose, melibiose, raffinose, GOS, GOS inulin, lactose, lactulose, beta-glucotriose B, cellobiose, Beneo P95 (Beneo-Orafti, Belgium), Raftilose P95 (Beneo-Orafti, Belgium), Raftilose Synergy 1 (Beneo-Orafti, Belgium), fructose, galactose, glucose, maltose, mannose, sucrose, the sources and preparation of which were as previously described [23].

Reconstituted skimmed milk (RSM) was prepared as a 10% (w/v) solution and autoclaved at 121°C for 10 minutes. Strains were inoculated into the RSM at 1% (v/v) and incubated for 72 hours at 37°C. Following the incubation period the pH of each culture was recorded and any change was adjusted by the change value of the negative control to identify the net pH change.

For aerobic growth, strains were inoculated as a 1% (v/v) inoculum in 5 ml of MRS and grown overnight aerobically at 37°C. Optical density (OD) readings were recorded at 0 hr and 24 hr.

The API-ZYM kit (bio-Merieux, France) was used to characterise the enzyme activity in whole bacterial cells. The tests were carried out in duplicate following the manufacturer’s instructions. Beta galactosidase activity was assayed in duplicate using OPNG disks (Sigma Aldrich, Co. Wicklow, Ireland) as per the manufacturer’s instructions.

### Bile salt resistance, low pH tolerance and EPS production

To assess the effect of increasing concentrations of porcine bile salts (Sigma Aldrich, Wicklow) and lowered pH on *L. ruminis* viability, modifications were made to MRS. For the bile salt assay MRS was supplemented with 0.25-5% (w/v) porcine bile salts. In the acid tolerance assay, the pH was reduced using acetic acid from pH 5.5 to 3.0 in step-wise pH 0.5 increments.

Exopolysaccharide production was analysed using modified MRS supplemented with 70% (v/v) of filter sterilised glucose, sucrose and lactose.

### Antibiotic resistance

Rifampicin and chloramphenicol were chosen as exemplars of broad-spectrum antibiotics. Each antibiotic was tested using sterile disks (Sigma Aldrich, Wicklow, Ireland) on MRS agar plates supplemented with each test strain. The disks were saturated with rifampicin (0.1-1 μg/ml) and chloramphenicol (1-4 μg/ml). The test plates containing the disks were then grown at 37°C for 48 hr. A strain was considered resistant if no zone of clearing was present surrounding the antibiotic disk.

### DNA extraction, 16S rRNA gene amplification and sequence analysis

DNA was extracted from bacterial isolates using the Sigma Genelute Bacterial genomic DNA kit (Arklow, Wicklow, Ireland). The primers used in this study are listed in Additional file 1. Universal primers 27 F and 1492R [23] were used to amplify the 16S rRNA gene in a 50 μl reaction mixture consisting of 45 μl Platinum High Fidelity Supermix (Invitrogen, USA), each primer at 25 μM, 20 ng of template DNA and water to make the reaction up to 50 μl. Amplification conditions for the PCR included an initial denaturation step of 94°C for 2 min, followed by 35 cycles of 94°C for 20 s, 52°C for 30 s, and 68°C for 2 min and a final extension step of 68°C for 10 min. PCR products were checked for size and purity on a 1% (w/v) agarose gel using gel electrophoresis. PCR products were purified with the QIAquick PCR purification kit (Qiagen, USA). DNA sequencing of the amplified 16S rRNA gene region was carried out by Beckmann Coulter Genomics (Takely, UK). Sequence alignments were performed using the ClustalW application in BioEdit [66]. MEGA (version 5) [67] was used to construct trees by using the neighbour-joining algorithm and the Kimura two-parameter substitution model. Branch support was measured by 1,000 replicate bootstrap tests for each analysis.

### Assessment of flagellum production and motility

*L. ruminis* cells were stained with a crystal violet-based flagellar stain (BD Diagnostics). The procedure was carried out as outlined by the manufacturer. Stained cells were then examined by light microscopy under oil immersion using 1,000X magnification, and images were captured using an Olympus DP50 camera attached to the microscope.

### Multi-locus sequence typing

The nucleotide sequences of the following genes were used for MLST analysis: *ftsQ, nrdB, parB*, *pheS*, *pstB* and *rpoA*. Primer pairs for each locus were designed using BioEdit [66]. An approximately 800 bp internal fragment of each gene was amplified, which allowed subsequent sequencing of an internal 600–760 bp fragment within each amplicon, using the primers specified in Additional file 1. Individual PCR products were sequenced (Beckman Coulter genomics, Takely, UK) and trimmed using Bioedit. Different allelic sequences, with at least one nucleotide difference per allele, were assigned arbitrary numbers. A combination of six alleles defined the allelic profile of each strain, and a unique allelic profile was designated with a sequence type (ST). Split decomposition analysis of the allelic profile data and individual alleles was performed using SplitsTree 4.8 [68]. Concatenated sequences (4,103 bp) of the loci (ordered as *ftsQ*, *nrdB*, *parB*, *pheS*, *pstB* and *rpoA*) were generated using the Sequence type Analysis and Re-combinatorial Tests (START2) software [69]. One thousand replicate neighbour-joining bootstrap trees were constructed using the Kimura 2-parameter method [70] in MEGA version 5 [67] to determine phylogeny. The relatedness of the isolates was assessed using START2; related STs were clustered in groups or lineages using BURST analysis. START2 was also used to determine the ratio of non-synonymous to synonymous polymorphisms (dN/dS ratio) for each locus [69]. Statistical comparisons were carried out using the maximum chi-square analysis application in the START2 package.

### Genome sequencing and comparative genomics

Sequencing was carried out for strains, S23 (human isolate), DPC 6832 (equine isolate), DPC 6830 (porcine isolate) and ATCC 27780 (bovine isolate) using the Illumina HiSeq 2000 reversible dye terminator system with read lengths of 101 bp. The functional assignment of predicted genes was performed using Metagene [71] to predict open reading frames (ORFs) and BLASTP was used forannotation against the NCBI non-redundant protein database [72]. Whole genome comparisons were made using the Artemis Comparison Tool (ACT) [73]. BLAST ring image generator (BRIG) [74] was used to create an image of whole genome comparisons.

QuartetS, which uses a reciprocal-best BLAST approach followed by 2-stage clustering, was used to predict orthologs. A core genome of 907 genes was identified. ClustalW [75] was used to create PHYLIP files from the amino acids of each core gene, which were imported into Clann (version 3.2.3) [76] to create a bootstrapped supertree.

### RNA isolation and RNA-seq

*L. ruminis* ATCC 27782 and DPC 6832 were cultured anaerobically at 37°C for 18 hours in 5 ml aliquots of MRS media (swimming cells) and also on MRS agar plates containing 0.5% (w/v) agar (swarming cells) and 2% (w/v) agar (stationary cells) for 48 hours. The broth cultures were centrifuged at 4°C to harvest the cells that were immediately resuspended in 10 ml of RNAprotect Bacteria Reagent (Qiagen, Germany). To each agar plate 10 ml of RNAprotect Bacteria Reagent was added and the cells gently harvested using sterile spreaders and removed from the plate using a wide-bore pipette tip into a fresh 50 mL Falcon tube. Each tube was centrifuged at 4,000 × g for 15 min at 4°C. Total RNA was isolated according to the protocol for Gram-positive bacteria outlined by the Roche High Pure Isolation kit (Roche, Indiana, USA), but with minor modifications. The lysozyme step was shortened and the concentration was increased to 100 mg/ml. Additionally, this step was also merged with a bead-beating step to ensure complete cell lysis; the cells were incubated for 60 min at 37°C while shaking at 1400 rpm in a 2 ml stock tube containing 0.1 mm zirconia beads in an Eppendorf Thermomixer. Contaminating DNA was removed with the Turbo DNA-free kit (Invitrogen, Dun Laoghaire, Ireland). The total RNA was ribo-depleted using the Gram-Positive Bacteria Ribo-Zero™ Magnetic Kit (Cambio Ltd., Cambridge, UK) and cleaned using the RNA Clean & Concentrator™-5 (Cambridge Biosciences, Cambridge, UK). The mRNA fragmentation, random-primed cDNA synthesis, adapter ligation, adapter-specific PCR amplification and pooling of the six tagged libraries into one pool were carried out by GATC Biotech (Konstanz, Germany).

Each sample was run on an Illumina HiSeq sequencer (GATC Biotech, Konstanz, Germany) to generate 101 bp length reads using paired-end sequencing. FastaQC was used to identify the quality of the RNA-seq reads from each treatment (www.bioinformatics.babraham.ac.uk). The Trimmomatic program was used to trim low-quality sections of reads [77,78]. Alignment of the reads to the complete genome of ATCC 27782 and the draft genome of DPC 6832 was carried out using Bowtie2 [79]. HTSeq-count and DESeq were utilised to assess differential gene expression between stationary, swimming and swarming *L. ruminis* cells [80,81].

### RT-PCR

RT-PCR was used to confirm differential expression of selected genes. The SensiFAST™ SYBR® No-ROX One-Step Kit (Bioline, myBio, Ireland) was used to generate cDNA and RT-PCR was performed according to the manufacturer’s specifications. The amplification temperature for all reactions was 55°C. The expression data generated for each gene and condition (stationary, swimming and swarming) for *L. ruminis* strains ATCC 27782 and DPC 6832 were normalised using the housekeeping gene *recA*. Following normalisation, fold differences were calculated using the following formula: fold change = 2^^(ΔΔCt)^. The standard deviation of the ΔCT was calculated from the standard deviations of the target and reference values using the formula: S.D. = (S_1_^2^ + S_2_^2^)^^0.5^. The resulting value was then added and subtracted to the ΔΔCT values to generate a range for the 2^^(ΔΔCt)^ values.

### Nucleotide sequences

This Whole Genome Shotgun sequences for *L. ruminis* strains, DPC 6832, S23, ATCC 27780 and DPC 6830 have been deposited at DDBJ/EMBL/GenBank under the accession numbers AWYA00000000, AWYB00000000, JHAJ00000000 and JHAB00000000, respectively. The versions described in this paper are versions AWYA01000000, AWYB01000000, JHAJ01000000 and JHAB01000000, respectively.

## Additional files

Additional file 1:
**Primers used in this study.**
Additional file 2:
**Neighbour-joining phylogenetic trees based upon 16S rRNA genes.**
Additional file 3:
**Sequence characteristics of the internal gene fragments used for multilocus sequence typing analysis.**
Additional file 4:
**Neighbour-joining phylogenetic trees for the MLST loci.**
Additional file 5:
**Prebiotic operon comparisons between ATCC 25644, ATCC 27782, S23 and DPC 6832.**
Additional file 6:
**BRIG comparison of ATCC 27782, ATCC 25644, S23 and DPC 6832.**
Additional file 7:
**Simulated gastric juice survival data for sixteen** ***L. ruminis*** **strains.**
Additional file 8:
**Technologically related phenotypic traits of the human, bovine, porcine and equine** ***L. ruminis*** **isolates.**
Additional file 9:
**Screening of equine** ***L. ruminis*** **isolates for their swarming phenotype in the presence of varying percentages of agar, the bio-surfactant Tween 80 and minimal carbohydrates.**
Additional file 10:
**Differentially expressed genes in swimming and swarming** ***Lactobacillus ruminis*** **ATCC 27782 cells.**
Additional file 11:
***Differentially expressed genes in swimming and swarming*** ***Lactobacillus ruminis*** **DPC6832 cells.**

## Abbreviations

FOS: Fructooligosaccharide
GOS: Galactooligosaccharide
GIT: Gastrointestinal tract
DP: Degree of polymerisation
LAB: Lactic acid bacteria
TLR: Toll-like receptor
ACT: Artemis comparison tool
BRIG: BLAST ring image generator
MLST: Multi locus sequence typing
SGJ: Simulated gastric juice
