# Supplementary material for: Lactobacillus ruminis strains cluster according to their mammalian gut source
Source: BMC Microbiol. 2015 Apr 1;15:80. doi: 10.1186/s12866-015-0403-y (PMC4393605; doi:10.1186/s12866-015-0403-y)
Supplement: Additional file 1: — Primers used in this study. [file 12866_2015_403_MOESM1_ESM.docx]

| **Additional file 1 Primers used in this study** | | | | |
| --- | --- | --- | --- | --- |
| **Primer** | **Sequence 5’-3’** | **Gene amplified** | **Size of amplicon (bp)** | **Source or reference** |
| 16S-Lru_F | ACCATGAACACCGCATGATGTTC | 16S rRNA | 849 | This study |
| 16S-Lru_R | TTCCATCTCTGGAATTGTCAGAAG |  |  |  |
| 27F | AGAGTTTGATCMTGGCTCAG | 16S rRNA | 1500 | (2) |
| 1492R | TACGGCACCTTGTTACGACTT |  |  |  |
| ftsQ-F | GTGCAGCACGTTGGACGATATCATC | *ftsQ* | 745 | This study |
| ftsQ-R | TTTTAGGATATGCGTAAGCTCCGACT |  |  |  |
| nrdB-F | AAGTTTTCGGAGGGCTGAC | *nrdB* | 733 | This study |
| nrdB-R | CCGTTTCCGACCTGAGAGAA |  |  |  |
| parB-F | CGGACTTGACGCATTATTCACTGAA | *parB* | 754 | This study |
| parB-R | GCTCTTGATTGAAACCTTCGTACTGA |  |  |  |
| pheS-F | GGACCTATTACTGAAGTGCTCCG | *pheS* | 839 | This study |
| pheS-R | TCCGGTCCAAGACCAAATGC |  |  |  |
| pstB-F | GACGTTCATCTGTACTATGGCAAA | *pstB* | 696 | This study |
| pstB-R | TTTGTTGTCCGGCGTCACAA |  |  |  |
| rpoA-F | CGCTTGAACGTGGCTATGGT | *rpoA* | 846 | This study |
| rpoA-R | CCAAGATCTGCCAACTTAGCC |  |  |  |
| rpsB-F | TCGTCGTTGGAACCCAAAGA | *rpsB* | 728 | This study |
| rpsB-R | AGTCTTCTTTACCTTCAACG |  |  |  |
| RT-PCR_1-F | AAGATCGGGAGTTTGTTGC | LRN_87/LRC_00640 | 82 | This study |
| RT-PCR_1-R | CCGAAAAGCTCATCTGAATC |  |  |  |
| RT-PCR_2-F | TCAAGCTTCAGGAAATCTGC | LRN_108/LRC_00780 | 219 | This study |
| RT-PCR_2-R | CCTGCTGAATATGTTTTGCC |  |  |  |
| RT-PCR_3-F | GGCGAAAGTTTGATGAAGAC | LRN_109/pfkB | 220 | This study |
| RT-PCR_3-R | GCGCATATGAACGATAGACC |  |  |  |
| RT-PCR_4-F | AGCCTGCACATCTCTTCTTC | LRN_110/LRC_00800 | 188 | This study |
| RT-PCR_4-R | GTTTTCAGCTTCCTTCCTTG |  |  |  |
| RT-PCR_5-F | GTCATGTCAAGGTTTTGCG | LRN_324/LRC_03250 | 218 | This study |
| RT-PCR_5-R | TGCTCCGAGAATAAGATTGC |  |  |  |
| RT-PCR_6-F | AGGGGAACGTACCGAAAAG | LRN_409/LRC_04370 | 113 | This study |
| RT-PCR_6-R | GCATGGTCCAAATCAATGTC |  |  |  |
| RT-PCR_7-F | TTATCGTCTCGGCTACCATC | LRN_520 | 163 | This study |
| RT-PCR_7-R | AATCATGTCCCTGCTTCTTG |  |  |  |
| RT-PCR_8-F | GACGCTTGCCTATCTTTCC | LRN_521 | 182 | This study |
| RT-PCR_8-R | CAGATCCGATCCAGAACAG |  |  |  |
| RT-PCR_9-F | GATGACCTCAGCCAAAAGC | LRN_561/LRC_05780 | 130 | This study |
| RT-PCR_9-R | CGTACGTGTCCAAGAAAACC |  |  |  |
| RT-PCR_10-F | CAGCAGCCAATTCAATACG | LRN_598/LRC_06170 | 103 | This study |
| RT-PCR_10-R | GCTGAGTTCGACATCCATC |  |  |  |
| RT-PCR_11-F | TGATGACGAACGCTTGAAC | LRN_933 | 110 | This study |
| RT-PCR_11-R | CTCTTCCCAATGCTGACTTG |  |  |  |
| RT-PCR_12-F | ACGTCGCAGCTATGAACAC | LRN_1405&1777/fliC | 159 | This study |
| RT-PCR_12-R | AACCACCGATTTGTGACTTC |  |  |  |
| RT-PCR_13-F | CAGGTTTGCGTATCAACAAG | LRN_1410/LRC_15700 | 165 | This study |
| RT-PCR_13-R | GAATGCTGTGAGTTTCGTTC |  |  |  |
| RT-PCR_14-F | CGAACGGTCAATACCAAATC | LRN_1655/LRC_18780 | 185 | This study |
| RT-PCR_14-R | GATCGGAACGAAAACATCAG |  |  |  |
| RT-PCR_15-F | GTGGCTTGTAATGCTATTCC | LRC_16260 | 96 | This study |
| RT-PCR_15-R | CTAACTGATTGTTTCGGCC |  |  |  |
| RecA-F | TTGGGAATCGTGTTCGTATC |  |  |  |
| RecA-R | TTCACCGGTCTTGGAAATC | *recA* | 156 | This study |
