# Supplementary material for: Lactobacillus ruminis strains cluster according to their mammalian gut source
Source: BMC Microbiol. 2015 Apr 1;15:80. doi: 10.1186/s12866-015-0403-y (PMC4393605; doi:10.1186/s12866-015-0403-y)
Supplement: Additional file 2: — Neighbour-joining phylogenetic trees based upon 16S rRNA genes. [file 12866_2015_403_MOESM2_ESM.docx]

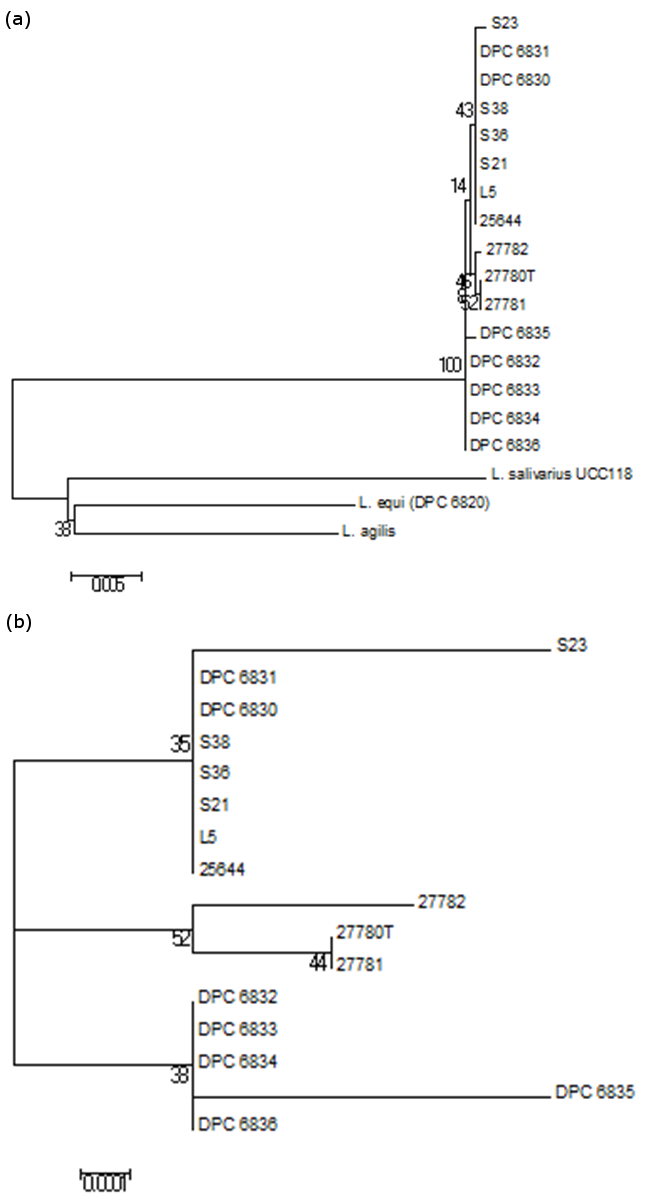


**Additional file 2 Neighbour joining phylogenetic tree** based upon (a) 16S rRNA genes sequences of *L. ruminis* isolates and selected other *Lactobacillus salivarius* clade species. (b) Neighbour joining phylogenetic tree for the *L. ruminis* isolates alone.
