# Supplementary material for: Lactobacillus ruminis strains cluster according to their mammalian gut source
Source: BMC Microbiol. 2015 Apr 1;15:80. doi: 10.1186/s12866-015-0403-y (PMC4393605; doi:10.1186/s12866-015-0403-y)
Supplement: Additional file 3: — Sequence characteristics of the internal gene fragments used for multilocus sequence typing analysis. [file 12866_2015_403_MOESM3_ESM.docx]

**Additional file 3 Sequence characteristics of the internal gene fragments used for multilocus sequence typing analysis**

| **Gene** | **Fragment analysed (nt)** | **Mean % GC of fragment** | **% GC of complete gene** | **Number of** | | **Nucleotide diversity per site** | **SSCF**  **(p value)** | **MCF**  **(p value)** |
| --- | --- | --- | --- | --- | --- | --- | --- | --- |
|  |  |  |  | **Alleles** | **Polymorphic sites** |  |  |  |
| *ftsQ* | 658 | 41.99 | 40.71 | 6 | 22 | 0.01008 | 263 (0.343) | 9 (1.000) |
| *nrdB* | 660 | 44.38 | 44.65 | 8 | 23 | 0.01096 | 1425 (0.106) | 16 (0.119) |
| *parB* | 673 | 46.23 | 45.27 | 6 | 22 | 0.00942 | 462 (0.658) | 13 (1.000) |
| *pheS* | 748 | 46.73 | 45.18 | 8 | 19 | 0.00806 | 1615 (0.160) | 16 (1.000) |
| *pstB* | 616 | 48.86 | 47.09 | 7 | 18 | 0.00930 | 622 (0.595) | 12 (1.000) |
| *rpoA* | 765 | 42.03 | 41.69 | 6 | 9 | 0.00356 | 241 (0.126) | 7 (1.000) |
