# Supplementary figures and images for: Lactobacillus ruminis strains cluster according to their mammalian gut source
Source: BMC Microbiol. 2015 Apr 1;15:80. doi: 10.1186/s12866-015-0403-y (PMC4393605; doi:10.1186/s12866-015-0403-y)

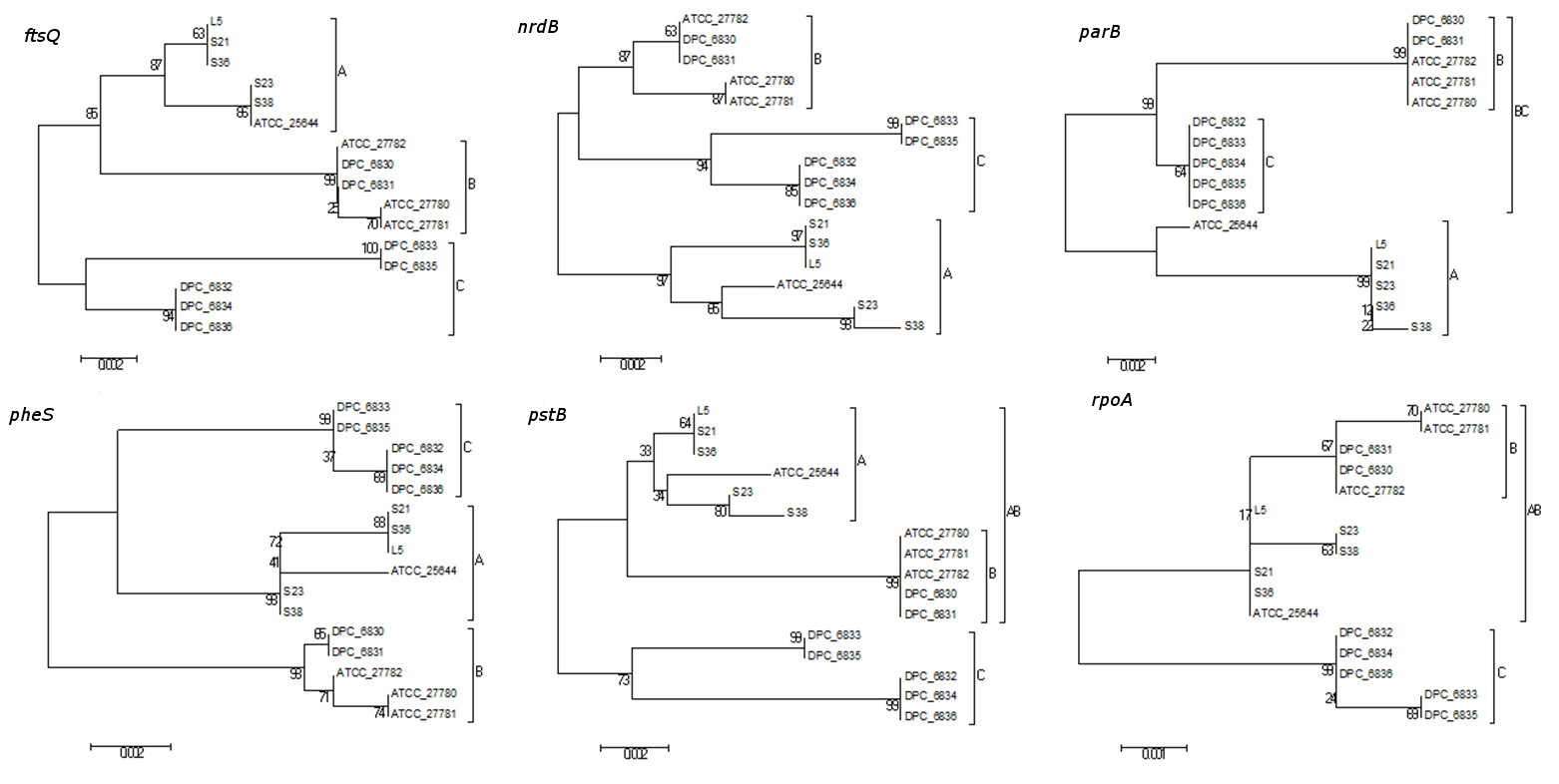


**Additional file 4 Neighbour-joining phylogenetic trees for the MLST loci indicated.**

Supplement: Additional file 4: — Neighbour-joining phylogenetic trees for the MLST loci. [file 12866_2015_403_MOESM4_ESM.docx]

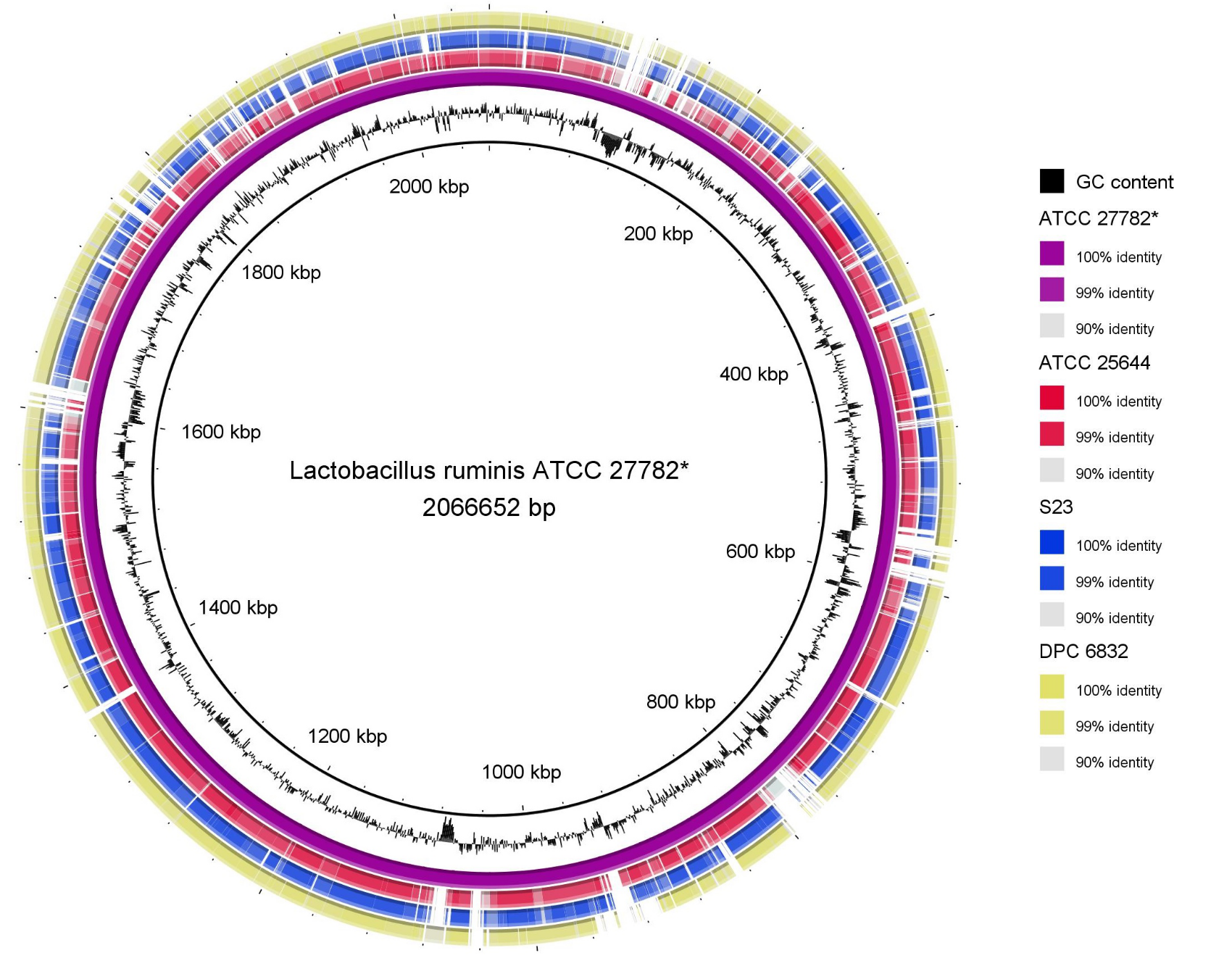


**Additional file 6 BRIG comparison** between ATCC 27782, ATCC 25644, S23 and DPC 6832.

Supplement: Additional file 6: — BRIG comparison of ATCC 27782, ATCC 25644, S23 and DPC 6832. [file 12866_2015_403_MOESM6_ESM.docx]

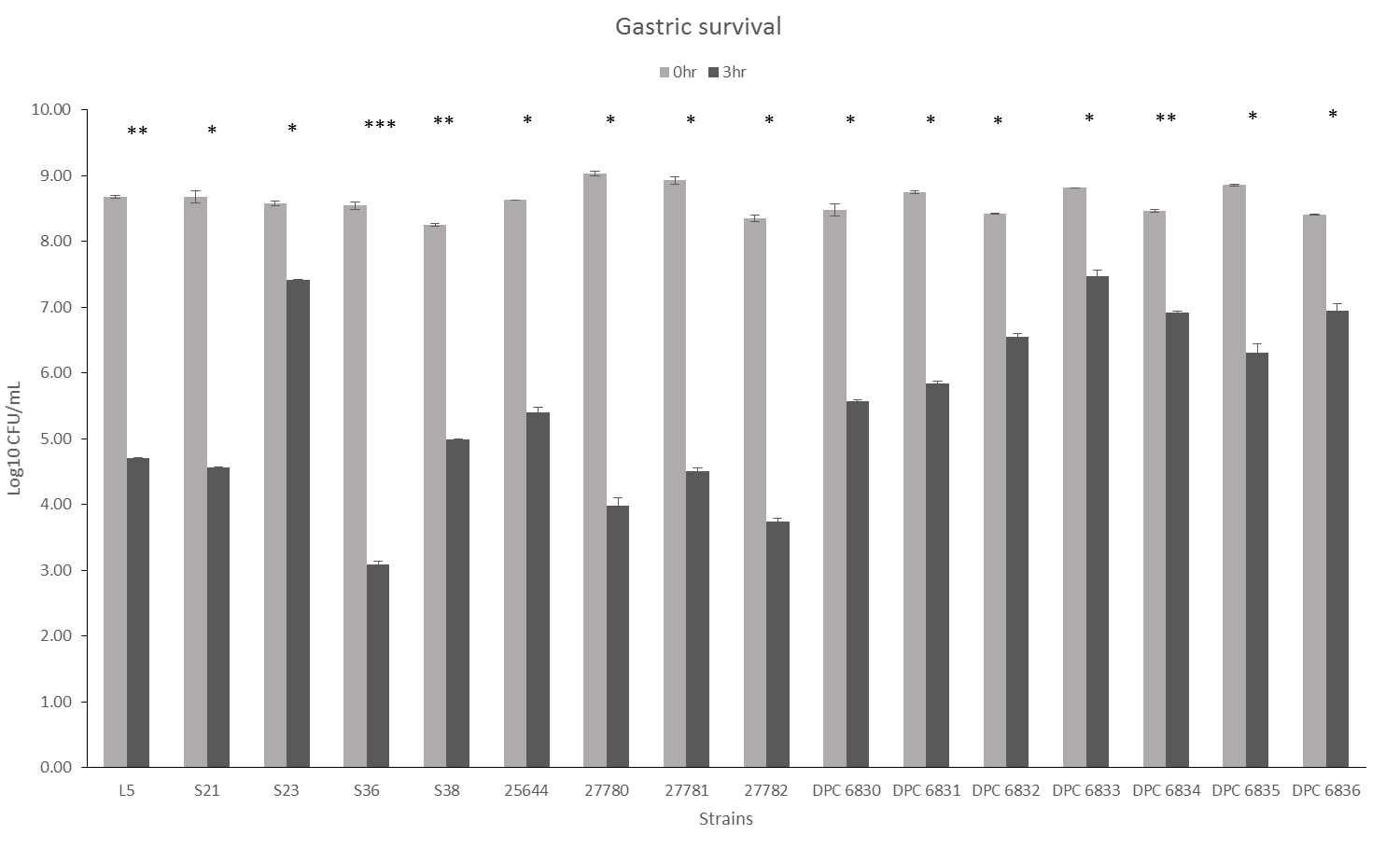


**Additional file 7 Simulated gastric juice survival data for sixteen *L. ruminis* strains**.

* P>0.05; ** P>0.01; *** P>0.001

Supplement: Additional file 7: — Simulated gastric juice survival data for sixteen L. ruminis strains. [file 12866_2015_403_MOESM7_ESM.docx]
