# Supplementary material for: Lactobacillus ruminis strains cluster according to their mammalian gut source
Source: BMC Microbiol. 2015 Apr 1;15:80. doi: 10.1186/s12866-015-0403-y (PMC4393605; doi:10.1186/s12866-015-0403-y)
Supplement: Additional file 5: — Prebiotic operon comparisons between ATCC 25644, ATCC 27782, S23 and DPC 6832. [file 12866_2015_403_MOESM5_ESM.docx]

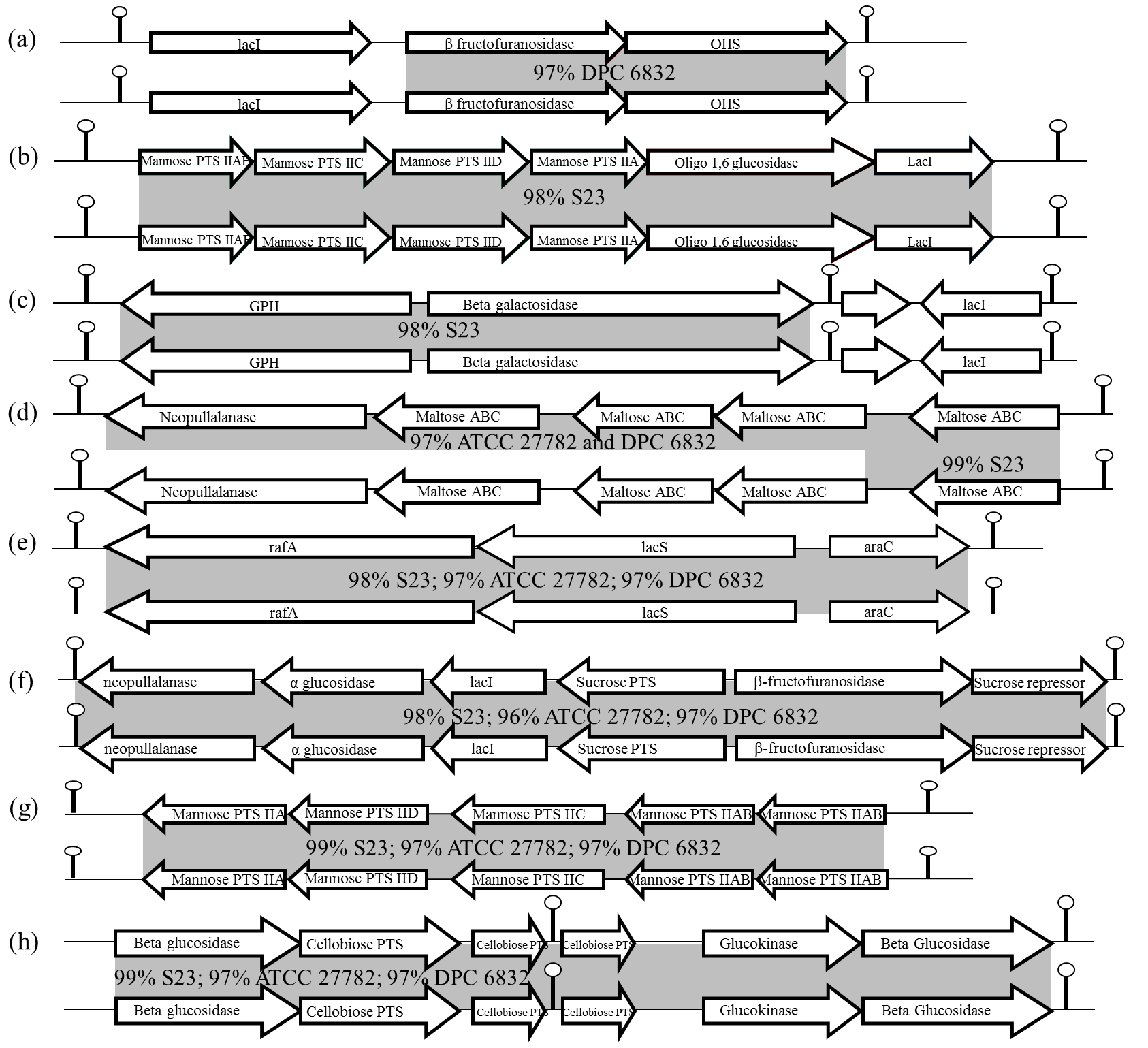


**Additional file 5 Prebiotic operon comparisons** between ATCC 25644, ATCC 27782, S23 and DPC 6832. (a) FOS operon (b) Mannose PTS operon 1 (c) Lactose operon 1 (*lacZ*1) (d) Maltose ABC operon (e) Raffinose operon (f) Sucrose PTS operon (g) Mannose PTS operon 3 (h) Cellobiose PTS operon
