# Supplementary material for: Lactobacillus ruminis strains cluster according to their mammalian gut source
Source: BMC Microbiol. 2015 Apr 1;15:80. doi: 10.1186/s12866-015-0403-y (PMC4393605; doi:10.1186/s12866-015-0403-y)
Supplement: Additional file 8: — Technologically related phenotypic traits of the human, bovine, porcine and equine L. ruminis isolates. [file 12866_2015_403_MOESM8_ESM.docx]

| Additional file 8 Technologically related phenotypic traits of the human, bovine, porcine and equine *L. ruminis* isolates | | | | | | | | | | | | | | | | | |
| --- | --- | --- | --- | --- | --- | --- | --- | --- | --- | --- | --- | --- | --- | --- | --- | --- | --- |
| **Tests** | **Conc./Variable** | **Human** | | | | | | **Bovine** | | | **Porcine** | | **Equine** | | | | |
|  |  | **L5** | **S21** | **S23** | **S36** | **S38** | **25644** | **27780** | **27781** | **27782** | **DPC 6830** | **DPC 6831** | **DPC 6832** | **DPC 6833** | **DPC 6834** | **DPC 6835** | **DPC 6836** |
| NaCl | 2% | + | + | + | + | + | + | + | + | + | + | + | + | + | + | + | + |
|  | 3% | + | + | + | + | + | + | + | + | + | + | + | + | + | + | + | + |
|  | 4% | + | + | + | + | + | + | + | + | + | + | + | + | + | + | - | - |
|  | 6% | - | - | - | - | - | - | - | - | - | - | - | - | - | - | - | - |
|  |  |  |  |  |  |  |  |  |  |  |  |  |  |  |  |  |  |
| Temp. | 4°C | - | - | - | - | - | - | - | - | - | - | - | - | - | - | - | - |
|  | 30°C | + | + | + | + | + | + | + | + | + | + | + | + | + | + | + | + |
|  | 37°C | + | + | + | + | + | + | + | + | + | + | + | + | + | + | + | + |
|  | 45°C | + | + | + | + | + | + | + | + | + | + | + | + | + | + | + | + |
|  | 55°C | - | - | - | - | - | - | - | - | - | - | - | - | - | - | - | - |
|  |  |  |  |  |  |  |  |  |  |  |  |  |  |  |  |  |  |
| Anaerobic | OD | 1.7 | 2 | 2.1 | 2.1 | 2.2 | 2.2 | 2.2 | 2.2 | 2.2 | 2.3 | 2.3 | 2.3 | 2.3 | 2.3 | 2.3 | 2.4 |
| Aerobic |  | 0 | 1.7 | 0.4 | 0.4 | 0.3 | 0.5 | 0.5 | 0.6 | 2.2 | 2.2 | 2.2 | 2 | 2.2 | 1.4 | 2.2 | 2.1 |
|  |  |  |  |  |  |  |  |  |  |  |  |  |  |  |  |  |  |
| Milk acidification^*^ | pH reduction | 1.6 | 1.4 | 2 | 1.6 | 2 | 1.2 | 2 | 2.2 | 2.2 | 1.9 | 2.2 | 1.4 | 1.5 | 0.2 | 1.8 | 1.3 |
