# Supplementary material for: Lactobacillus ruminis strains cluster according to their mammalian gut source
Source: BMC Microbiol. 2015 Apr 1;15:80. doi: 10.1186/s12866-015-0403-y (PMC4393605; doi:10.1186/s12866-015-0403-y)
Supplement: Additional file 9: — Screening of equine L. ruminis isolates for their swarming phenotype in the presence of varying percentages of agar, the bio-surfactant Tween 80 and minimal carbohydrates. [file 12866_2015_403_MOESM9_ESM.docx]

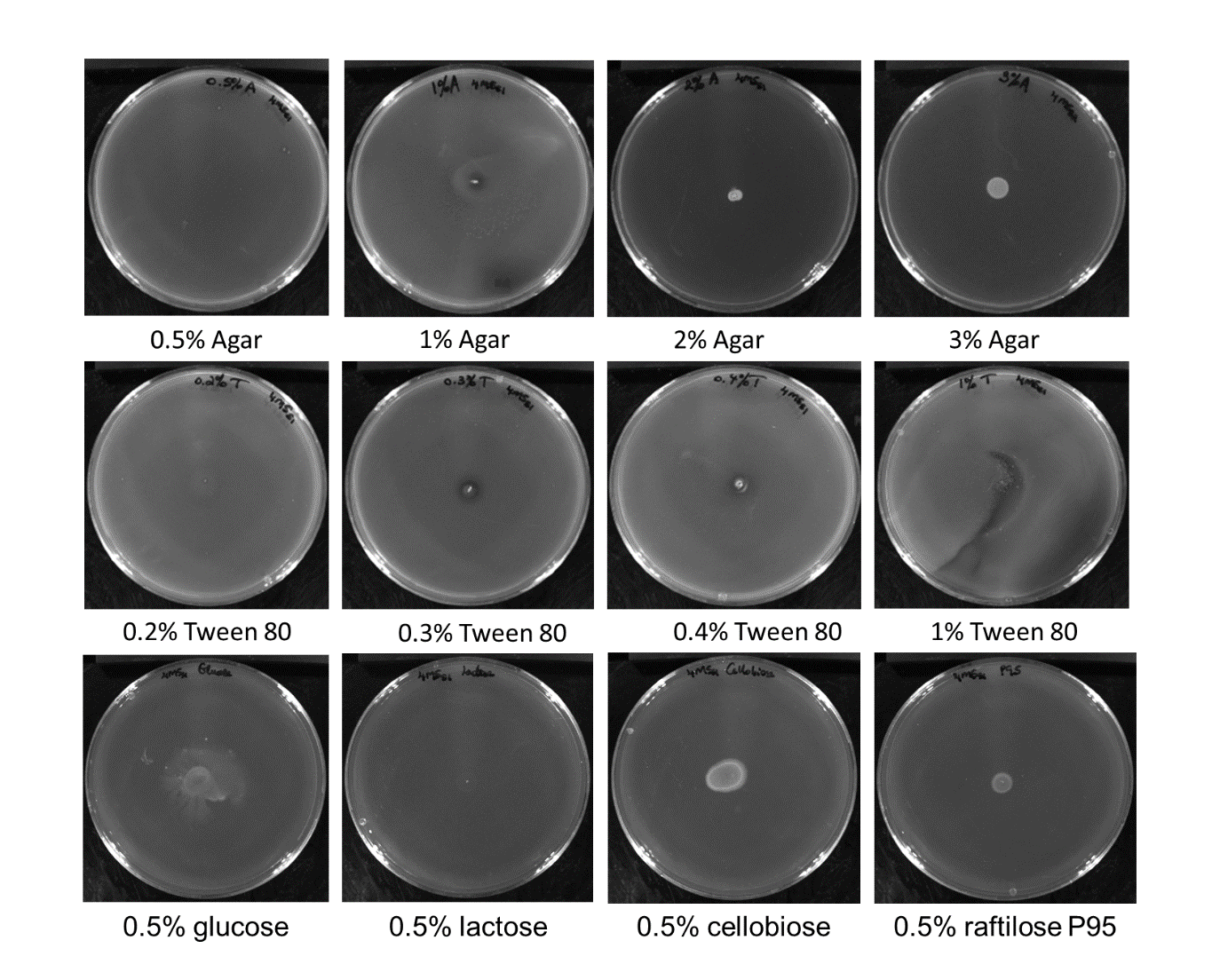


**Additional file 9 Screening of equine *L. ruminis* isolates for their swarming phenotype** in the presence of varying percentages of agar, the bio-surfactant Tween 80 and minimal carbohydrates. Note - All of the images above are from the equine DPC 6833 but are typical of the results seen with the other equine and porcine strains tested. The colour of the images was inverted as to emphasise the colonies and swarms, the colonies therefore appear as white and un-colonised agar appears dark grey.
