# Supplementary material for: Lactobacillus ruminis strains cluster according to their mammalian gut source
Source: BMC Microbiol. 2015 Apr 1;15:80. doi: 10.1186/s12866-015-0403-y (PMC4393605; doi:10.1186/s12866-015-0403-y)
Supplement: Additional file 10: — Differentially expressed genes in swimming and swarming Lactobacillus ruminis ATCC 27782 cells. [file 12866_2015_403_MOESM10_ESM.docx]

| **Additional file 10 Statistically significantly differentially expressed genes in swimming and swarming *Lactobacillus ruminis* ATCC 27782 cells** | | | | | | |
| --- | --- | --- | --- | --- | --- | --- |
| **id** | **ATCC 27782 Swimming vs Stationary^a^** | **pval** | **ATCC 27782 Swarming vs Stationary^b^** | **pval** | **Fold change** | **GENBANK FUNCTION** |
| LRC_18740 | -5.45 | * | 0.15 | >0.05 | 48.51 | maltose/maltodextrin ABC transporter binding protein |
| LRC_18780 | 5.11 | *** | -0.48 | >0.05 | 48.06 | PTS system sucrose-specific transporter subunit IIABC |
| LRC_0703l | -1.54 | >0.05 | 3.18 | * | 26.40 | tRNA-Tyr |
| LRC_02110 | 3.41 | * | -1.31 | >0.05 | 26.29 | DegV family protein |
| LRC_0638a | 5.59 | * | 0.94 | >0.05 | 25.19 | tRNA-Arg |
| LRC_16560 | 4.62 | ** | 0.30 | >0.05 | 19.94 | ribose-5-phosphate isomerase |
| LRC_07250 | -3.74 | * | 0.33 | >0.05 | 16.84 | hypothetical protein |
| serS | -3.25 | * | 0.79 | >0.05 | 16.48 | seryl-tRNA synthetase |
| LRC_1388a | 4.50 | ** | 0.47 | >0.05 | 16.36 | tRNA-Glu |
| LRC_15450 | 3.78 | * | -0.15 | >0.05 | 15.25 | glutaredoxin |
| LRC_05410 | 3.70 | * | -0.18 | >0.05 | 14.68 | toxin/antitoxin system, Toxin component |
| LRC_19580 | 3.35 | * | -0.49 | >0.05 | 14.36 | transcriptional regulator |
| LRC_18160 | 4.47 | ** | 0.71 | >0.05 | 13.53 | isochorismatase family protein |
| lytR | 4.73 | ** | 1.00 | >0.05 | 13.26 | LytR family transcriptional regulator |
| LRC_03890 | 4.11 | ** | 0.44 | >0.05 | 12.71 | NlpC/P60 |
| LRC_0373f | 4.73 | ** | 1.07 | >0.05 | 12.62 | tRNA-Thr |
| LRC_05420 | 2.82 | * | -0.83 | >0.05 | 12.59 | toxin/antitoxin system, Antitoxin component |
| LRC_255m | 4.55 | * | 0.94 | >0.05 | 12.22 | tRNA-Pro |
| LRC_08030 | -3.37 | * | 0.23 | >0.05 | 12.19 | hypothetical protein |
| LRC_0425a | 4.01 | ** | 0.46 | >0.05 | 11.73 | tRNA-Thr |
| LRC_04960 | 3.27 | * | -0.06 | >0.05 | 10.08 | hypothetical protein |
| LRC_18260 | 3.24 | * | -0.08 | >0.05 | 10.00 | AraC family transcriptional regulator |
| LRC_07100 | -3.11 | * | 0.21 | >0.05 | 9.98 | hypothetical protein |
| LRC_18150 | 4.07 | * | 0.83 | >0.05 | 9.47 | hypothetical protein |
| LRC_07510 | -2.88 | * | 0.28 | >0.05 | 8.96 | hypothetical protein |
| rpsL | 3.94 | ** | 0.79 | >0.05 | 8.92 | 30S ribosomal protein S12 |
| rplC | 3.13 | * | -0.02 | >0.05 | 8.89 | 50S ribosomal protein L3 |
| **LRC_05780** | 4.04 | ** | 0.89 | >0.05 | 8.85 | hypothetical protein |
| LRC_255n | 4.39 | ** | 1.28 | >0.05 | 8.65 | tRNA-Pro |
| LRC_11220 | 3.51 | * | 0.41 | >0.05 | 8.57 | hypothetical protein |
| LRC_17850 | 3.17 | * | 0.09 | >0.05 | 8.46 | glycosyltransferase |
| LRC_18790 | 3.56 | * | 0.53 | >0.05 | 8.16 | sucrose-6-phosphate hydrolase |
| rpsJ | 3.22 | * | 0.29 | >0.05 | 7.65 | 30S ribosomal protein S10 |
| LRC_12520 | 3.56 | * | 0.63 | >0.05 | 7.62 | 30S ribosomal protein S15 |
| LRC_17160 | 3.25 | * | 0.37 | >0.05 | 7.36 | D-Ala-teichoic acid biosynthesis protein |
| LRC_0373e | 5.27 | * | 2.52 | >0.05 | 6.72 | tRNA-Glu |
| LRC_17840 | 3.53 | * | 0.78 | >0.05 | 6.71 | hypothetical protein |
| LRC_02780 | 3.14 | * | 0.40 | >0.05 | 6.65 | 50S ribosomal protein L4 |
| LRC_00560 | 4.04 | ** | 1.31 | >0.05 | 6.64 | Deoxyguanosine kinase |
| LRC_06960 | 3.54 | * | 0.82 | >0.05 | 6.59 | glycosyltransferase |
| rplK | 3.52 | * | 0.81 | >0.05 | 6.56 | 50S ribosomal protein L11 |
| s6 | 3.38 | * | 0.68 | >0.05 | 6.50 | 30S ribosomal protein S6 |
| LRC_00520 | 3.56 | * | 0.86 | >0.05 | 6.49 | cytidine deaminase |
| rplV | 2.86 | * | 0.16 | >0.05 | 6.47 | 50S ribosomal protein L22 |
| rpsG | 3.30 | * | 0.67 | >0.05 | 6.22 | 30S ribosomal protein S7 |
| rpsS | 2.81 | * | 0.19 | >0.05 | 6.12 | 30S ribosomal protein S19 |
| rplB | 2.99 | * | 0.39 | >0.05 | 6.08 | 50S ribosomal protein L2 |
| rpl23p | 3.19 | * | 0.59 | >0.05 | 6.05 | 50S ribosomal protein L23 |
| LRC_0419b | 5.35 | *** | 2.76 | >0.05 | 6.05 | tRNA-Ala |
| ccpA | 2.84 | * | 0.29 | >0.05 | 5.88 | Catabolite control protein A |
| LRC_18800 | 3.05 | * | 0.50 | >0.05 | 5.87 | Sucrose operon repressor |
| LRC_16260 | 3.70 | * | 1.16 | >0.05 | 5.84 | hypothetical protein |
| LRC_06030 | 3.67 | * | 1.22 | >0.05 | 5.49 | CAAX family protease |
| infA | 3.12 | * | 0.67 | >0.05 | 5.45 | translation initiation factor IF-1 |
| LRC_14830 | 3.90 | * | 1.47 | >0.05 | 5.39 | hypothetical protein |
| rpoA | 2.84 | * | 0.42 | >0.05 | 5.36 | DNA-directed RNA polymerase subunit alpha |
| ezrA | 2.98 | * | 0.60 | >0.05 | 5.20 | septation ring formation regulator |
| LRC_19820 | 2.99 | * | 0.73 | >0.05 | 4.78 | ribonuclease P |
| LRC_18350 | 3.24 | * | 1.03 | >0.05 | 4.62 | multidrug/hemolysin transport system ATP-binding protein |
| LRC_01390 | 2.88 | * | 0.74 | >0.05 | 4.40 | UDP-galactopyranose mutase |
| rplA | 3.14 | * | 1.05 | >0.05 | 4.26 | 50S ribosomal protein L1 |
| LRC_1447a | 0.85 | * | 2.91 | * | 4.19 | 5S ribosomal RNA |
| **LRC_17530** | 2.98 | * | 0.97 | >0.05 | 4.03 | transposase |
| LRC_16270 | 3.37 | * | 1.37 | >0.05 | 3.99 | Secreted LysM-domain containing protein |
| LRC_0703p | 2.86 | * | 0.94 | >0.05 | 3.77 | tRNA-Cys |
| LRC_12720 | 3.15 | * | 1.27 | >0.05 | 3.68 | hypothetical protein |
| LRC_0703k | 3.91 | * | 2.05 | >0.05 | 3.62 | tRNA-Phe |
| LRC_06490 | 2.84 | * | 1.00 | >0.05 | 3.58 | hypothetical protein |
| trnA | 3.67 | * | 1.86 | >0.05 | 3.51 | tRNA-Ala |
| LRC_01380 | 2.83 | * | 1.14 | >0.05 | 3.22 | oligosaccharide translocase |
| LRC_12530 | 3.53 | * | 1.93 | >0.05 | 3.03 | 30S ribosomal protein S20 |
| LRC_0703q | 3.08 | * | 1.59 | >0.05 | 2.80 | tRNA-Leu |
| LRC_10980 | 2.82 | * | 1.37 | >0.05 | 2.75 | hypothetical protein |
| LRC_255k | 3.63 | * | 2.79 | * | 1.79 | tRNA-Leu |

a – negative values indicate a down-regulation of swimming cells

b – negative values indicate a down-regulation of swarming cells
