# Supplementary material for: Lactobacillus ruminis strains cluster according to their mammalian gut source
Source: BMC Microbiol. 2015 Apr 1;15:80. doi: 10.1186/s12866-015-0403-y (PMC4393605; doi:10.1186/s12866-015-0403-y)
Supplement: Additional file 11: — Differentially expressed genes in swimming and swarming Lactobacillus ruminis DPC6832 cells. [file 12866_2015_403_MOESM11_ESM.docx]

| **Additional file 11 Statistically significantly differentially expressed genes in swimming and swarming *Lactobacillus ruminis* DPC6832 cells** | | | | | | |
| --- | --- | --- | --- | --- | --- | --- |
| **id** | **DPC 6832 Swimming vs Stationary^a^** | **pval** | **DPC 6832 Swarming vs Stationary^b^** | **pval** | **Fold change** | **GENBANK FUNCTION** |
| LRN_108 | -5.11 | *** | 2.66 | * | 218 | DeoR fructose transcriptional regulator |
| LRN_109 | -3.94 | ** | 3.62 | ** | 189 | 1-phosphofructokinase |
| LRN_110 | -2.93 | * | 4.29 | ** | 149 | PTS_system,_fructose_specific_IIABC_component |
| LRN_0409 | -4.62 | *** | 2.28 | >0.05 | 120 | hypothetical_protein_LRU_02075 |
| LRN_0721 | -3.50 | ** | 1.71 | >0.05 | 37 | phosphoenolpyruvate_carboxykinase_(ATP) |
| LRN_0087 | -3.36 | ** | 1.81 | >0.05 | 36 | hypothetical_protein_HMPREF0542_11617 |
| LRN_1651 | -2.66 | * | 2.31 | >0.05 | 31 | maltose\|maltodextrin_ABC_superfamily_ATP_binding_cassette |
| LRN_1460 | 2.48 | * | -2.47 | * | 31 | LysM_domain_protein |
| LRN_1355 | -2.06 | >0.05 | 2.64 | * | 26 | ArsR_family_transcriptional_regulator |
| LRN_329 | -1.97 | >0.05 | 2.68 | * | 25 | myosin-cross-reactive_antigen |
| LRN_1539 | -2.81 | * | 1.79 | >0.05 | 24 | aldose_1-epimerase |
| LRN_376 | -3.54 | ** | 0.99 | >0.05 | 23 | transposase_ISSoc7 |
| LRN_521 | 0.06 | >0.05 | 4.49 | *** | 22 | MFS Transporter Beta fructofuranosidase |
| LRN_1540 | -3.00 | * | 1.35 | >0.05 | 20 | aldose_1-epimerase |
| LRN_1620 | -2.86 | * | 1.38 | >0.05 | 19 | sugar_ABC_superfamily_ATP_binding_cassette |
| LRN_520 | -0.74 | >0.05 | 3.39 | * | 18 | beta-fructofuranosidase |
| LRN_1692 | -3.19 | * | 0.74 | >0.05 | 15 | endonuclease\|exonuclease\|phosphatase_family_protein |
| LRN_1708 | 2.74 | * | -1.15 | >0.05 | 15 | membrane_protein |
| LRN_0692 | -2.58 | * | 1.25 | >0.05 | 14 | hypothetical_function_DUF299 |
| LRN_1751 | 3.07 | * | -0.56 | >0.05 | 12 | hypothetical_protein_LRC_02660 |
| LRN_1784 | 2.81 | * | -0.56 | >0.05 | 10 | transposase |
| LRN_0324 | 2.50 | * | -0.86 | >0.05 | 10 | hypothetical_protein_HMPREF0542_10780 |
| LRN_1236 | 2.71 | * | -0.57 | >0.05 | 10 | inositol-phosphate phosphatase |
| LRN_1587 | -2.43 | * | 0.69 | >0.05 | 9 | Peroxiredoxin (PRX) family |
| LRN_1452 | 2.60 | * | -0.49 | >0.05 | 9 | flagellar_basal_body_rod_protein |
| LRN_1764 | 2.46 | * | -0.62 | >0.05 | 8 | transposase |
| LRN_1338 | -2.41 | * | 0.63 | >0.05 | 8 | ferritin,_Dps_family_protein |
| LRN_1527 | 3.39 | * | 0.35 | >0.05 | 8 | putative_peptide\|deacylase |
| LRN_1800 | 3.05 | * | 0.19 | >0.05 | 7 | transposase |
| LRN_1337 | -2.45 | * | 0.36 | >0.05 | 7 | cytochrome_b5 |
| LRN_1756 | -0.10 | >0.05 | 2.70 | * | 7 | hypothetical_protein_LGG_01889 |
| LRN_1574 | 2.88 | * | 0.10 | >0.05 | 7 | hypothetical_protein_LRC_17840 |
| LRN_1451 | 2.41 | * | -0.33 | >0.05 | 7 | flagellar_basal-body_rod_protein_FlgC |
| LRN_0058 | 0.30 | >0.05 | 2.83 | * | 6 | pyruvate_formate-lyase_activating_enzyme |
| LRN_526 | 2.77 | * | 0.27 | >0.05 | 6 | MFS_transporter DBSA oxidoreductase |
| LRN_1265 | 2.34 | * | -0.12 | >0.05 | 6 | (3R)-hydroxyacyl-[acyl_carrier_protein]_dehydratase |
| LRN_1746 | 3.13 | * | 0.71 | >0.05 | 5 | ferulic_acid_esterase |
| LRN_1788 | 3.98 | ** | 1.59 | >0.05 | 5 | transposase |
| LRN_1218 | 2.67 | * | 0.35 | >0.05 | 5 | F0F1_ATP_synthase_subunit_A |
| LRN_1217 | 2.53 | * | 0.22 | >0.05 | 5 | ATP_synthase_F0_sector_subunit_C |
| LRN_0056 | 0.32 | >0.05 | 2.64 | * | 5 | pyruvate formate-lyase activating enzyme |
| LRN_0756 | 2.57 | >0.05 | 0.31 | >0.05 | 5 | phosphatidylserine_decarboxylase_proenzyme_2 |
| LRN_1424 | 2.72 | * | 0.49 | >0.05 | 5 | chemotaxis_protein_methyltransferase |
| LRN_1425 | 2.50 | * | 0.27 | >0.05 | 5 | chemotaxis_response_regulator_protein-glutamate_methylesterase |
| LRN_1700 | 3.40 | ** | 1.17 | >0.05 | 5 | inosine guanoisine nucleoside hydrolase |
| LRN_1426 | 2.47 | * | 0.29 | >0.05 | 5 | CheW chemotaxis protein |
| LRN_0783 | 3.11 | * | 1.01 | >0.05 | 4 | hypothetical_protein_HMPREF0542_10419 |
| LRN_0784 | 2.52 | * | 0.47 | >0.05 | 4 | arginyl-tRNA_synthetase |
| LRN_1331 | 2.37 | * | 0.33 | >0.05 | 4 | VanZ_family_protein |
| LRN_1590 | 2.80 | * | 0.77 | >0.05 | 4 | HIT_family_protein |
| LRN_1523 | 2.66 | * | 0.65 | >0.05 | 4 | D-alanine--poly(phosphoribitol)_ligase_subunit_1 |
| LRN_1422 | 2.40 | * | 0.41 | >0.05 | 4 | chemotaxis_protein_CheC |
| LRN_1438 | 2.53 | * | 0.54 | >0.05 | 4 | flagellar_biosynthesis_protein_FliO |
| LRN_1658 | 2.52 | * | 0.53 | >0.05 | 4 | endonuclease\|exonuclease\|phosphatase_family_protein |
| LRN_1576 | 2.77 | * | 0.82 | >0.05 | 4 | LysE_family_L-lysine_permease |
| LRN_1420 | 2.77 | * | 0.84 | >0.05 | 4 | chemotaxis_signal_transduction_protein_CheW |
| LRN_0741 | 2.56 | * | 0.64 | >0.05 | 4 | GMP_reductase |
| LRN_0933 | 5.15 | *** | 3.24 | * | 4 | hypothetical_protein_HMPREF0542_11529 |
| LRN_1421 | 2.47 | * | 0.56 | >0.05 | 4 | chemotaxis_protein_CheY |
| LRN_1423 | 2.38 | * | 0.48 | >0.05 | 4 | histidine_kinase |
| LRN_0932 | 5.17 | *** | 3.32 | * | 4 | transposase,_ISSmi4 |
| LRN_1215 | 2.40 | * | 0.55 | >0.05 | 4 | ATP_synthase_F1_sector_delta_subunit |
| LRN_1437 | 2.47 | * | 0.71 | >0.05 | 3 | flagellar_biosynthetic_protein_FliP |
| LRN_1789 | 2.47 | * | 0.75 | >0.05 | 3 | MutR family transcriptional regulator |
| LRN_1020 | 2.82 | * | 1.11 | >0.05 | 3 | transposase |
| LRN_1522 | 2.79 | * | 1.09 | >0.05 | 3 | D-alanine-poly(phosphoribitol)_ligase_subunit_2 |
| LRN_1659 | 3.28 | ** | 1.68 | >0.05 | 3 | PTS_family_glucose_porter,_IICBA_component |
| LRN_1416 | 2.39 | * | 0.83 | >0.05 | 3 | flagellar_motor_switch_protein |
| LRN_0277 | 2.32 | * | 0.81 | >0.05 | 3 | 50S_ribosomal_protein_L23 |
| LRN_1455 | 3.02 | * | 1.72 | >0.05 | 2 | methyl-accepting_chemotaxis_protein |
| LRN_0070 | 1.41 | >0.05 | 2.70 | * | 2 | membrane protein |
| LRN_1454 | 2.70 | * | 1.42 | >0.05 | 2 | flagellar_motor_protein_A |
| LRN_1410 | 1.81 | >0.05 | 3.04 | * | 2 | flagellin |
| LRN_0032 | 2.47 | * | 1.24 | >0.05 | 2 | 30S_ribosomal_protein_S6 |
| LRN_1777 | 1.89 | >0.05 | 3.09 | * | 2 | flagellin |
| LRN_1405 | 1.70 | >0.05 | 2.89 | * | 2 | flagellin |
| LRN_0466 | 3.22 | * | 2.10 | >0.05 | 2 | xanthine_phosphoribosyltransferase |
| LRN_1521 | 2.38 | * | 1.30 | >0.05 | 2 | D-alanine_transfer_protein_DltD |
| LRN_1762 | 3.28 | * | 2.23 | >0.05 | 2 | N-acetyltransferase |
| LRN_1401 | 1.47 | >0.05 | 2.50 | * | 2 | hypothetical_protein_HMPREF0542_12012 |
| LRN_0467 | 1.96 | >0.05 | 2.60 | * | 2 | xanthine_permease |
| LRN_1384 | 2.45 | * | 2.04 | >0.05 | 1 | methyl_accepting_chemotaxis_protein |
| LRN_0904 | 3.22 | * | 3.13 | * | 1 | hypothetical_protein_ANHS_1530 |

a – negative values indicate a down-regulation of swimming cells

b – negative values indicate a down-regulation of swarming cells
